# *S*-nitrosylation of a receptor-like cytoplasmic kinase regulates plant immunity

**DOI:** 10.1126/sciadv.adk3126

**Published:** 2024-03-15

**Authors:** Beimi Cui, Qiaona Pan, Wenqiang Cui, Yiqin Wang, Verity I. P. Loake, Shuguang Yuan, Fengquan Liu, Gary J. Loake

**Affiliations:** ^1^ Department of Plant Pathology, Key Laboratory of Agricultural Microbiology, College of Agriculture, Guizhou University, Guiyang, 550025, China.; ^2^ Institute of Plant Protection, Jiangsu Academy of Agricultural Sciences, Jiangsu Key Laboratory for Food Quality and Safety-State Key Laboratory Cultivation Base of Ministry of Science and Technology, Nanjing, 210014, China.; ^3^ Institute of Molecular Plant Sciences, School of Biological Sciences, University of Edinburgh, Edinburgh, EH9 3BF, UK.; ^4^ University of Chinese Academy of Sciences, Beijing, 100049, China.; ^5^ Institute of Biomedicine and Biotechnology, Shenzhen Institutes of Advanced Technology, Chinese Academy of Sciences, Shenzhen, 518055, China.; ^6^ Institute of Genetics and Developmental Biology, Chinese Academy of Sciences, Beijing, 100101, China.; ^7^ Faculty of Medicine, South Kensington Campus, Imperial College London, London, SW7 2AZ, UK.; ^8^ Centre for Engineering Biology, School of Biological Sciences, University of Edinburgh, Edinburgh, EH9 3BF, UK.

## Abstract

Perception of pathogen/microbial-associated molecular patterns (P/MAMPs) by plant cell surface receptors leads to a sustained burst of reactive oxygen species (ROS), a key feature of P/MAMP-triggered immunity (PTI). Here we report that P/MAMP recognition leads to a rapid nitrosative burst, initiating the accumulation of nitric oxide (NO), subsequently leading to *S*-nitrosylation of the receptor-like cytoplasmic kinase (RLCK), botrytis-induced kinase 1 (BIK1), at Cys^80^. This redox-based, posttranslational modification, promotes the phosphorylation of BIK1, subsequently resulting in BIK1 activation and stabilization. Further, BIK1 *S*-nitrosylation increases its physical interaction with RBOHD, the source of the apoplastic oxidative burst, promoting ROS formation. Our data identify mechanistic links between rapid NO accumulation and the expression of PTI, providing insights into plant immunity.

## Teaser

*S*-nitrosylation of the plant pathogen receptor, BIK1, promotes its phosphorylation, activation, and stabilization.

## INTRODUCTION

Changes in cellular redox status are a conspicuous feature of immune signaling cascades throughout eukaryotes (*1*, *2*). In plants, recognition of pathogen/microbial-associated molecular patterns (P/MAMPs), including flagellin (or its derived epitope, flg22) and elongation factor Tu (or its derived epitope, elf18), by the membrane-located pattern recognition receptors, FLS2 and EFR, respectively, leads to P/MAMP-triggered immunity (PTI) (*3*, *4*).

Both FLS2 and EFR form ligand-induced complexes with the somatic embryogenesis receptor kinase (SERK) isoform, BRI1-associated kinase 1 (BAK1/SERK3), essential for downstream signaling (*5*–*7*). In addition, these receptor-coreceptor complexes also require the presence of the receptor-like cytoplasmic kinase, botrytis-induced kinase 1 (BIK1), which is released on ligand binding triggering a battery of downstream immune responses (*8*–*13*). For example, enhanced calcium influx through Ca^2+^-permeable channel proteins including reduced hyperosmolality-induced [Ca^2+^]_i_ increase 1.3 and cyclic nucleotide-gated channel 4 (*14*, *15*), engagement of mitogen-activated protein kinase signaling cascades (*16*, *17*), and a rapid, marked and transient burst of apoplastic reactive oxygen species (ROS) (*18*, *19*) predominantly derived from the reduced form of nicotinamide adenine dinucleotide phosphate (NADPH) oxidase, respiratory burst oxidase homolog D (RBOHD) (*20*).

Collectively, the deployment of this myriad of immune-related responses establishes PTI, restricting attempted pathogen infection. A rapid nitrosative burst, resulting in the production of nitric oxide (NO) and derived reactive nitrogen intermediates, is also engaged in response to P/MAMPs (*21*, *22*). However, the potential functions of these small, redox-active molecules in PTI remain largely unexplored.

## RESULTS

**Fig. 1.**
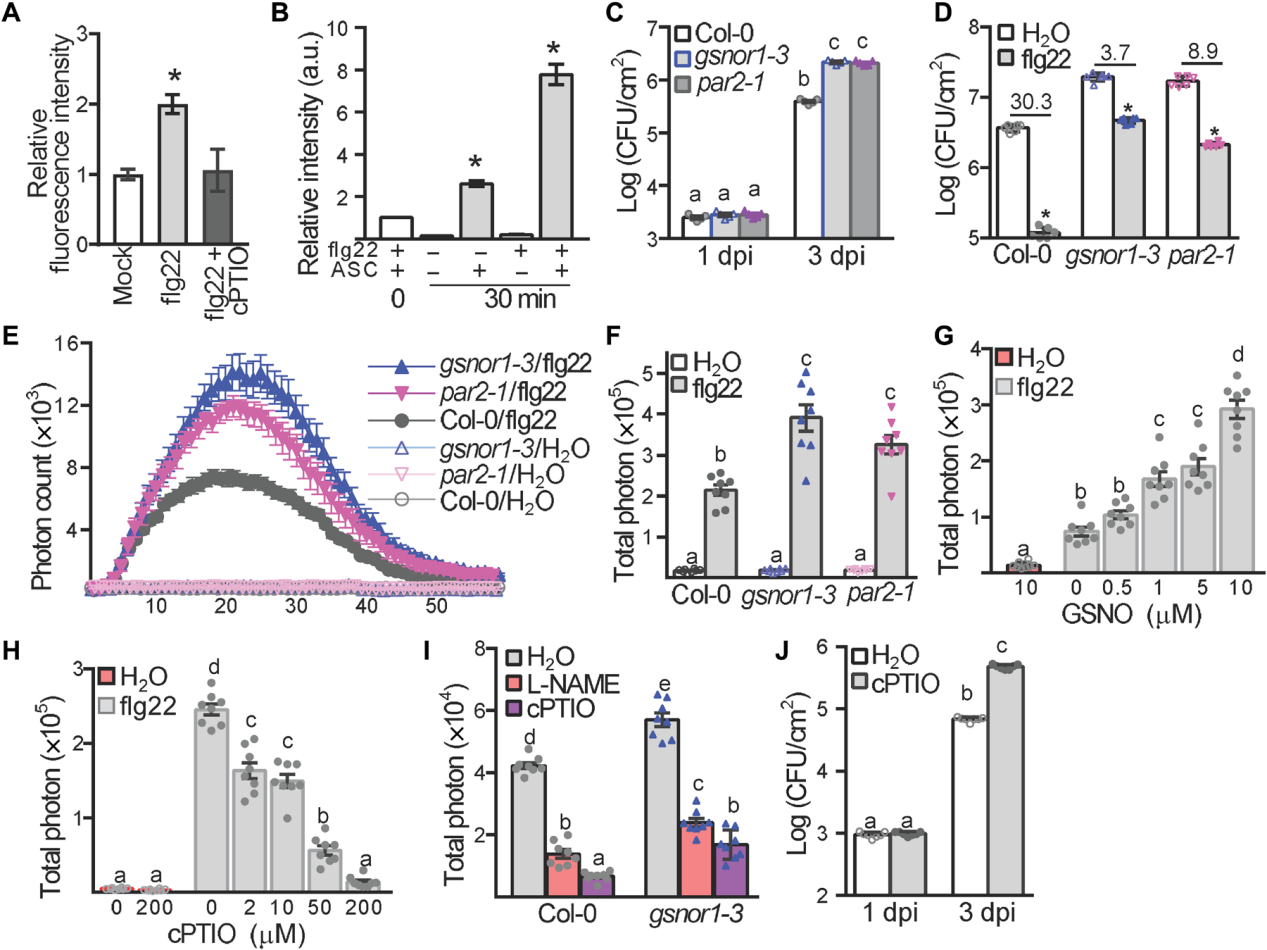
NO is required for flg22-induced ROS production in *Arabidopsis*. (**A**) NO levels were determined using the NO-sensitive probe DAF-FM DA. Roots of *Arabidopsis* seedlings were treated with 1 μM flg22 with or without 200 μM cPTIO for 10 min followed by quantification of DAF-FM DA staining. *n* = 7 (*P* < 0.05, *t* test). (**B**) Total protein *S*-nitrosylation (SNO) levels were determined by BST followed by quantification of the corresponding Western blot. Ten-day-old wild-type Col-0 seedlings were treated with 1 μM flg22 (+) or mock (−) and then subjected to the BST, followed by signal quantification. Asc, Ascorbate. (**C**) Titer of *Pst* DC3000 *hrcC^−^* was assessed. *n* = 6, *P* < 0.05 [one-way analysis of variance (ANOVA)]. (**D**) Titer of *Pst* DC3000 was assessed at 3 days post-inoculation (dpi) after 24 hours pretreatment with 1 μM flg22 or H_2_O. *n* = 6 (*P < 0.05 compared to the corresponding values of each H_2_O treatment). The number listed is the mean of the bacterial titer represents the fold change of H_2_O pretreatment compared to flg22 pretreatment. (**E** and **F**) Time course (E) and total photon counts (F) of flg22- or H_2_O- triggered ROS production. *n* = 8, *P* < 0.05 (one-way ANOVA). (**G** and **H**) Total photon counts were calculated in Col-0 after treatment with flg22 plus the indicated concentrations of GSNO (G) or cPTIO (H). *n* = 8 (one-way ANOVA; *P* < 0.05). (**I**) Total photon counts were calculated after treatment with flg22 plus 200 μM cPTIO or 200 μM L-NAME. *n* = 8 (one-way ANOVA; *P* < 0.05). (**J**) Titer of *Pst* DC3000 *hrcC^−^*. Col-0 plants were pretreated with 200 μM cPTIO for 24 hours and then spray inoculation with *Pst* DC3000 *hrcC^−^*. *n* = 7 (one-way ANOVA; *P* < 0.05). All experiments in this figure were repeated three times with similar results. a.u., arbitrary units.

To gain mechanistic insights into the possible role(s) of NO in PTI, we first explored NO production in *Arabidopsis* upon flagellin peptide flg22 treatment. Following application of this well-established P/MAMP, a rapid nitrosative burst was detected using the NO-sensitive reporter, diaminofluorescein-FM diacetate (DAF-FM DA) (*23*), within 10 min post-flg22 treatment (Fig. 1A and fig. S1A). However, the application of the NO scavenger, 2-4-carboxyphenyl-4,4,5,5-tetramethylimidazoline-1-oxyl-3-oxide (cPTIO), or an inhibitor of mammalian NO synthase, NG-nitro-l-arginine methyl ester (L-NAME) (*24*), largely removed this signal (Fig. 1A and fig. S1A), consistent with previous observations (*25*–*28*), indicating that NO might play important roles in PTI. A similar result was obtained with the bacterial pathogen strain *Pseudomonas syringae* pv. *tomato* (*Pst*) DC3000 *hrcC^−^* (*Pst* DC3000 *hrcC^−^*), deficient in type III secretion (*29*), which specifically induced PTI (fig. S1B). To establish whether this flg22-triggered nitrosative burst was dependent on flg22 recognition by FLS2, we compared NO production between wild-type Col-0 plants and a *fls2* line insensitive to this P/MAMP (*3*). After flg22 treatment, a notable nitrosative burst detected by DAF-FM DA was observed in wild-type Col-0 plants but not in the *fls2* mutant (fig. S1C). To determine whether the observed flg22-triggered, FLS2-dependent NO accumulation was associated with an increase in total cellular *S*-nitrosylation, an NO-dependent posttranslational modification, we performed a biotin switch technique (BST) (*30*), which specifically replaces a NO moiety covalently attached to a target protein with a biotin tag, which can subsequently be detected with an antibiotin antibody. Our results showed a notable increase in total protein *S*-nitrosylation after flg22 treatment (Fig. 1B).

Total cellular *S*-nitrosylation in *Arabidopsis* is controlled by *S*-nitrosoglutathione reductase 1 (GSNOR1), which metabolizes the natural NO donor, *S*-nitrosoglutathione (GSNO) (*31*, *32*). Therefore, to asses a potential function for *S*-nitrosylation in PTI, we challenged the *GSNOR1* loss-of-function mutants *gsnor1-3* and *Arabidopsis* paraquat resistant mutant *par2-1*, alleles of *gsnor1* (*31*, *33*) with *Pst* DC3000 *hrcC^−^*. Determination of the titer of *Pst* DC3000 *hrcC^−^* in leaves of the challenged *Arabidopsis* lines revealed that resistance against *Pst* DC3000 *hrcC^−^* was compromised in both *gsnor1-3* and *par2-1* plants (Fig. 1C). To confirm and extend these findings, we next pretreated both *gsnor1-3* and *par2-1* lines with flg22, to trigger the development of PTI, and then subsequently challenged these plants with *Pst* DC3000. Flg22 triggered the development of PTI in wild-type plants, leading to increased resistance against *Pst* DC3000; however, this response was diminished in the *gsnor1-3* and *par2-1* lines (Fig. 1D). Thus, dysregulation of *S*-nitrosylation compromises flg22-triggered, FLS2-dependent PTI.

To determine the molecular basis of this phenomenon, we examined well-established flg22-triggered outputs associated with PTI. Expression of flg22-induced receptor-like kinase 1 (*FRK1*), together with callose deposition scored by aniline blue staining, both markers of PTI (*6*, *7*, *14*), was reduced in the *gsnor1-3* mutant in contrast to that of the wild-type Col-0 plants (fig. S1, D and E). Counterintuitively, flg22-triggered ROS production was significantly enhanced in the *gsnor1-3* and *par2-1* lines (Fig. 1, E and F).

To further evaluate the role of NO in the P/MAMP-triggered ROS burst, we determined this response in the NO-overproducing (*nox1*) mutant (*34*). A highly increased flg22-triggered ROS burst was observed in the *nox1* mutant relative to wild-type Col-0 plants (fig. S2, A and B). *S*-nitrosylation can be promoted by applying well-established NO donors, including GSNO or sodium nitroprusside (SNP) (*23*). We therefore tested whether NO is required for flg22-mediated ROS production by using these donors. Adding a relatively low concentration of either GSNO (0.5 to 10 μM) or SNP (5 μM) markedly enhanced the flg22-triggered oxidative burst in a dose-dependent manner in wild-type Col-0 plants (Fig. 1G and fig. S2, C to E). However, a relatively high GSNO concentration (50 μM) inhibited the flg22-triggered ROS burst (fig. S2F), consistent with previous data suggesting accumulated NO at the later stages of a resistance protein triggered hypersensitive response, inhibits the RBOHD-dependent oxidative burst, curbing the immune response (*25*).

In contrast, the rapid flg22-triggered ROS burst was compromised following application of either the NO scavenger cPTIO or L-NAME (*24*), in both wild-type Col-0 and *gsnor1-3* plants (Fig. 1, H and I, and fig. S2G). Thus, demonstrating that NO is required for flg22-triggered ROS production. Similar results were observed in response to an alternative P/MAMP, elf18 (*4*), *Pst* DC3000, or *Pst* DC3000 *hrcC^−^* (figs. S3 and S4). In addition, using cPTIO compromised P/MAMP-based resistance to *Pst* DC3000 *hrcC^−^* (Fig. 1J). Together, our findings show that NO is a key regulator of ROS production during PTI.

**Fig. 2.**
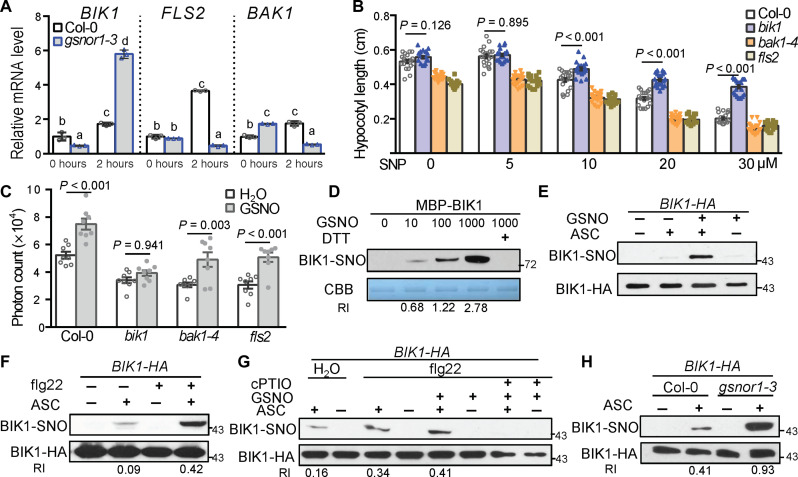
BIK1 is *S*-nitrosylated during PTI. (**A**) Relative transcript levels of *BIK1*, *FLS2*, and *BAK1* in inoculated Col-0 and *gsnor1-3* lines treated with 200 nM flg22 for 0 and 2 hours were determined by quantitative reverse transcription polymerase chain reaction (qRT-PCR). Relative expression levels were normalized to *UBQ10*, and the expression of each gene in Col-0 for 0 hours was set as 1. Values are mean ± SE, *n* = 3, and different letters indicate significant differences at *P* < 0.05, one-way ANOVA. (**B**) Hypocotyl length of indicated genotype upon given concentrations of SNP treatment for 5 days. Values are mean ± SE, *n* = 20, and *P* values as shown in figure are from two-tailed *t* test analysis. (**C**) Total photon counts were calculated in Col-0, *bik1*, *fls2*, and *bak1-4* over 40 min following treatment with 200 nM flg22 together with either H_2_O or 2 μM GSNO. Data are mean ± SE, *n* = 8 biologically intendent leaf discs and *P* values as shown are from two-tailed *t* test analysis. (**D**) Detection of BIK1-SNO in vitro. Recombinant MBP-BIK1 was exposed to a range of GSNO micromolar concentrations, subjected to the BST, and interrogated by Western blot analysis using an anti-biotin antibody. Protein loading was visualized by Coomassie brilliant blue (CBB) staining. (**E** to **H**) Detection of BIK1-SNO formation in vivo. Total protein extracts from *BIK1-HA* expressing plants were subjected to BST after exposure to the indicated molecules with 10 μM GSNO or 1 μM flg22 for 15 min. The bottom indicates total BIK1-HA loading detected by an anti-HA antibody. RI, relative intensity of BIK1-SNO formation compared to the corresponding BIK1-HA level. Three Biological repeats were performed with similar results.

We hypothesized that a central component of the PTI machinery might be a target of NO. To identify this potential target, we first examined the expression of *FLS2*, *BAK1*, and *BIK1*, key components of PTI signaling (*35*). The accumulation of *FLS2* and *BAK1* transcripts were both reduced in the *gsnor1-3* line relative to wild-type Col-0 plants upon flg22 treatment. In contrast, *BIK1* expression was enhanced (Fig. 2A). *fls2* and *bak1-4* mutants exhibited a NO-inhibited hypocotyl elongation phenotype comparable to wild-type Col-0 plants. Conversely, *bik1* mutants showed relative insensitivity to SNP treatment (Fig. 2B and fig. S5A). Further, the GSNO-mediated P/MAMP-triggered ROS burst was impaired in the *bik1* mutant relative to either wild-type Col-0, *bak1-4*, or *fls2* plants (Fig. 2C and fig. S5B). In aggregate, these results suggest that the observed NO-potentiated ROS burst during PTI is dependent on BIK1.

Since the predominant route for the transfer of NO bioactivity is *S*-nitrosylation (*36*, *37*), we next determined whether BIK1 is a target for *S*-nitrosylation. Thus, recombinant BIK1 was exposed to the natural NO donor, GSNO, at concentrations typically used to score for this modification in vitro (*25*) and monitored for the formation of BIK1 S-nitrosylation (BIK1-SNO) using the BST (*30*). BIK1 was *S*-nitrosylated in a concentration-dependent fashion by GSNO (Fig. 2D and fig. S5C). Furthermore, the addition of dithiothreitol (DTT) notably reduced the level of BIK1-SNO formation (Fig. 2D), consistent with the presence of a reversible thiol modification. The natural NO donor, Cys-NO, also *S*-nitrosylated BIK1 in vitro (fig. S5D).

To explore the possible *S*-nitrosylation of BIK1 in vivo following exposure to GSNO, endogenous proteins from hemagglutinin (HA)–tagged BIK1 (*BIK1-HA*) expressing plants were subjected to the BST and biotinylated proteins purified with streptavidin beads (*30*). Subsequently, these proteins were immuno-blotted with an anti-HA antibody. BIK1 was found to be *S*-nitrosylated in response to GSNO in vivo (Fig. 2E). In a similar fashion, either flg22 or *Pst* DC3000 *hrcC^−^* also promoted in vivo *S*-nitrosylation of BIK1 (Fig. 2F and fig. S5E). In addition, flg22 and GSNO in combination increased the level of BIK1-SNO, and application of the NO scavenger, cPTIO, compromised flg22- and GSNO-induced BIK1-SNO formation (Fig. 2G). We next determined the extent of BIK1-SNO formation in the *gsnor1-3* line, which exhibits a marked increase in this modification (*31*). Basal BIK1-SNO was observed in wild-type Col-0 plants, and BIK1-SNO was notably induced in *gsnor1-3* line compared with Col-0 (Fig. 2H and fig. S5F). Addition of flg22 induced BIK1-SNO formation both in Col-0 and *gsnor1-3* line (fig. S5F). Together, these results show that BIK1 is *S*-nitrosylated during PTI.

**Fig. 3.**
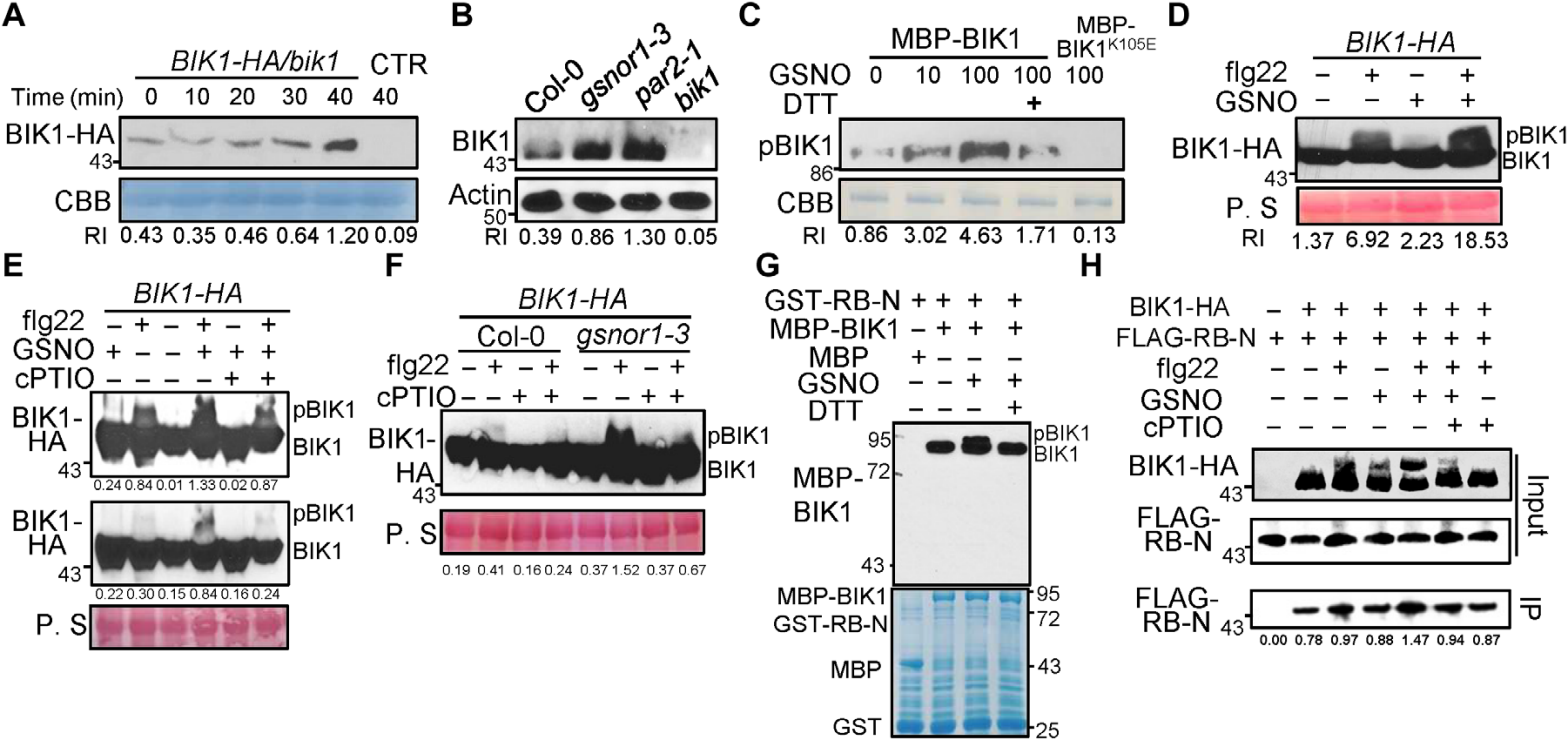
NO controls BIK1 stability and activity. (**A**) BIK1 abundance was detected. Total protein extracted from *BIK1-HA* expressing lines or Col-0 (CTR) was subjected to Western blotting after treatment with GSNO at the indicated times. CBB staining served as a loading control. (**B**) Total protein extracts from 10-day-old seedlings were subjected to Western blot using a BIK1 antibody. Actin was used as a loading control, and *bik1* mutants were used as a negative control. (**C**) BIK1 kinase activity was determined in vitro by examining its autophosphorylation. Recombinant maltose binding protein (MBP)–BIK1 was subjected to a kinase activity assay following treatment with the indicated concentrations of GSNO or 100 μM DTT. CBB staining was used as a loading control. (**D** to **F**) Flg22-induced pBIK1 was detected. Levels of phosphorylated BIK1 (pBIK1) in *BIK1-HA* expressing lines were detected by mobility shift assay using an anti-HA immunoblot after treatment with 1 μM flg22 with 10 μM GSNO or 200 μM cPTIO for 15 min. RI, relative intensity [top lane band compared to corresponding bottom lane band in (D)]. The top and bottom of (E) is from the same blot with a longer and a shorter exposure time, respectively. (**G**) GST pull-downs were performed to measure complex formation between MBP-BIK1 and GST-RB-N. GST-RB-N immobilized on glutathione sepharose was incubated with MBP or GSNO- or DTT-pretreated MBP-BIK1, and washed beads were subjected to an anti-MBP immunoblot. Input proteins are shown by CBB. (**H**) Co-IP assays of BIK1 and RBOHD-N. FLAG-RB-N construct was transiently expressed in protoplasts derived from *BIK1-HA* expressing plants for 16 hours and then treated with 1 μM flg22, 10 μM GSNO, or 200 μM cPTIO for 15 min. Total proteins (input) were subjected to immunoprecipitation with anti-HA agarose followed by immunoblot using anti-FLAG. All experiments were performed three times with similar results. P. S, Ponceau S.

The stability of BIK1 is important for its role during PTI (*35*, *38*–*40*). To test whether NO regulates BIK1 stability, we used NO donor GSNO and SNP, and both of them increased the abundance of BIK1, as scored by an anti-HA antibody (Fig. 3A and fig. S6A). In addition, BIK1 exhibited increased abundance in both *gsnor1-3* and *par2-1* mutants, which show increased global *S*-nitrosylation, compared with wild-type Col-0 plants (Fig. 3B). Informatively, in the presence of cycloheximide, which inhibits de novo protein synthesis (*38*), the NO donor GSNO increased BIK1 abundance (fig. S6B), suggesting that NO may inhibit BIK1 degradation. To confirm and extend these findings, we used MG132, a proteasome inhibitor which reduces the degradation of ubiquitin-conjugated proteins by binding to the catalytic sites of the proteasome and blocking its activity thereby preventing the degradation of ubiquitinated proteins (*41*). In the presence of MG132, BIK1 abundance increased to a level close to that established by GSNO. Further, BIK1 abundance was also increased in *gsnor1-3* plants (fig. S6C). Consistent with the evidence that BIK1 is rate limiting for PTI responses (*12*, *14*, *15*, *35*, *39*), flg22 triggered an increase in ROS production in transgenic plants that exhibited enhanced abundance of BIK1 (fig. S6, D and E). Together, these findings show that an increase in endogenous NO enhances BIK1 stability, with increased BIK1 abundance promoting the flg22-triggered ROS burst.

The kinase activity of BIK1 is critical for PTI signaling and engagement of the oxidative burst (*11*, *12*, *35*, *40*). We therefore determined whether NO regulates BIK1 kinase activity. Using an in vitro kinase assay (*11*), we found that GSNO enhanced BIK1 autophosphorylation in vitro, and this could be reversed by addition of the SNO-reducing agent, DTT, but GSNO could not induce autophosphorylation in a phopho-mutant version BIK1^K105E^ (Fig. 3C), which lacked kinase activity (*10*, *11*). We found that NO induced *S*-nitrosylation of BIK1^K105E^ (fig. S6F), demonstrating that a kinase-dead BIK1 variant does not affect NO-based modification of BIK1. Similar to flg22-triggered phosphorylated BIK1 analysis by mobility shift Western blot assay in vivo (*11*, *39*), GSNO and, to a lesser extent, SNP induced BIK1 activation in a similar fashion (fig. S7, A and B). Further, we determined the consequence of co-application of GSNO to flg22-triggered BIK1 phosphorylation. GSNO potentiated flg22-triggered BIK1 activation (Fig. 3D), and cPTIO diminished this response (Fig. 3E). In addition, using cPTIO reduced BIK1-SNO upon flg22 treatment (fig. S7C). Further, flg22-induced BIK1 phosphorylation was also enhanced in both *gsnor1-3* and *par2-1* plants compared with wild-type Col-0 (fig. S7D and Fig. 3F), and this was diminished in the presence of cPTIO (Fig. 3F). In addition, the application of λ protein phosphatase also reversed flg22- and GSNO-induced formation of phosphorylated BIK1 (pBIK1) (fig. S7E). To explore whether NO could also potentiate pBIK1 formation in response to other P/MAMPS, we determined the response to elf18. GSNO increased elf18-induced pBIK1 levels, and this response was compromised by cPTIO (fig. S8). Together, these results suggest that NO is required for optimal phosphorylation of BIK1 upon P/MAMP perception.

BIK1 plays a critical role in the P/MAMP triggered activation of the oxidation burst by interacting with and subsequently phosphorylating the NADPH oxidase, RBOHD (*8*, *10*). We therefore next tested whether *S*-nitrosylation of BIK1 affects its association with RBOHD. Pull-down assays in vitro revealed that the N-terminal domain of RBOHD interacts with BIK1 and that the association between pBIK1 and N-RBOHD was enhanced by GSNO (Fig. 3G). Next, we confirmed this direct/indirect physical interaction of pBIK1 with RBOHD after GSNO treatment by co-immunoprecipitation (co-IP) experiments in *Arabidopsis* protoplasts by transiently expressing the FLAG-RBOHD N terminus in *BIK1-HA* overexpression lines. GSNO promoted the interaction of BIK1 with RBOHD in vivo in a similar fashion to flg22, while GSNO together with flg22 further increased the interaction, which was reduced by cPTIO (Fig. 3H and fig. S9A). Similarly, addition of NO donor SNP also promoted interaction between BIK1 and RBOHD in yeast based on the Y2H assay (fig. S9B). Subsequently, we determined whether this GSNO-promoted interaction between BIK1 and RBOHD resulted in increased phosphorylation of RBOHD. Addition of GSNO promoted BIK1-dependent phosphorylation of RBOHD in vitro (fig. S9C). Collectively, these data indicate that GSNO promotes the interaction of BIK1 with RBOHD, and this enhanced interaction results in increased phosphorylation of RBOHD by BIK1.

**Fig. 4.**
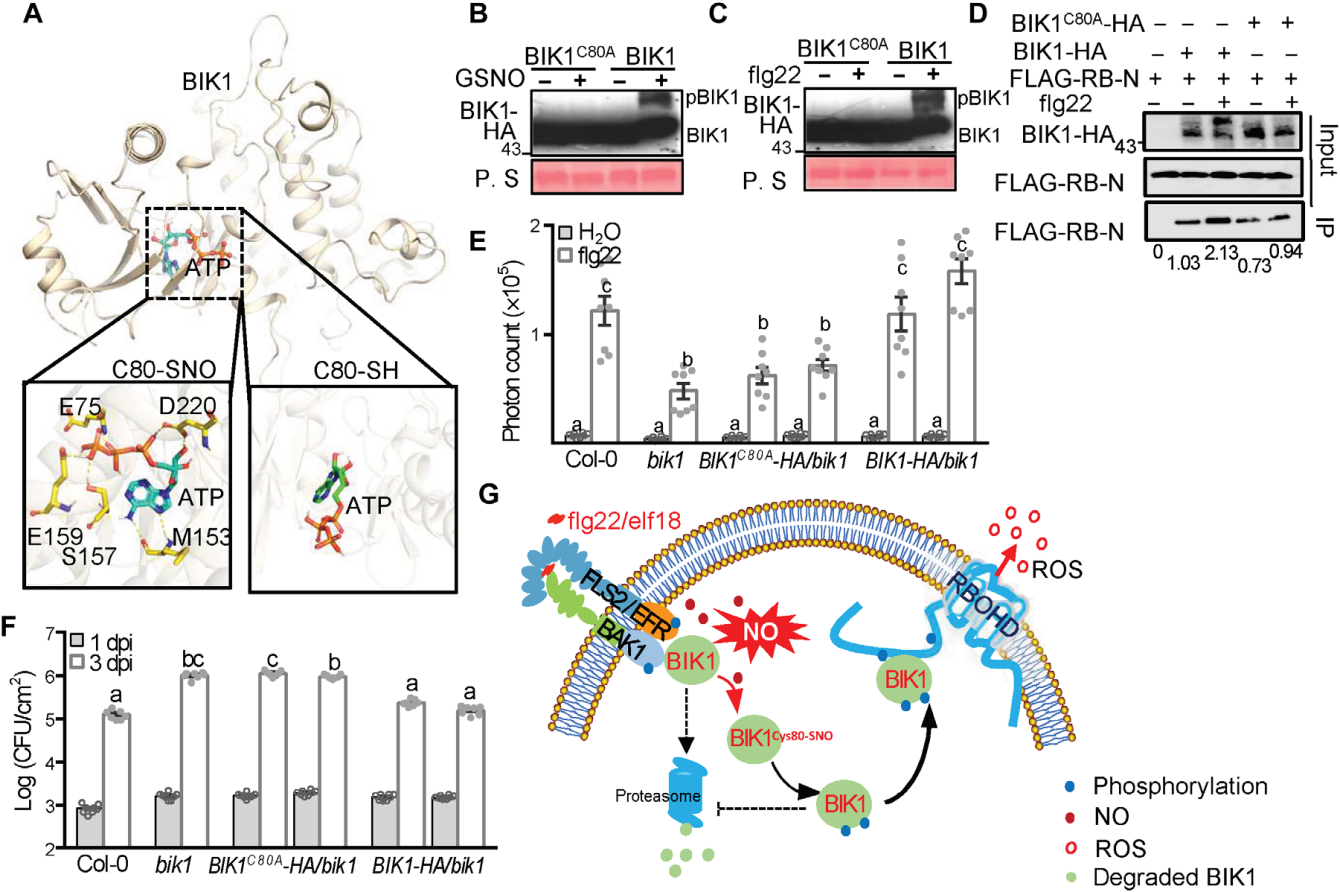
BIK1 Cys^80^ is required for PTI signaling. (**A**) Structural comparison of de-nitrosylated BIK1(C80-SH) or *S*-nitrosylated BIK1 (C80-SNO) with respect to their complexes with ATP. (**B** and **C**) GSNO- or flg22-induced pBIK1 was detected. Transgenic *Arabidopsis* Col-0 lines expressing either *BIK1-HA* or *BIK1^C80A^-HA* were treated with 10 μM GSNO (B) or 1 μM nM flg22 (C) for 15 min and subsequently subjected to a mobility shift assay using an immunoblot using an anti-HA antibody. Ponceau S staining of proteins was used as a loading control (bottom). (**D**) Co-IP assays of BIK1 or BIK1^C80A^ and RBOHD-N. Flag-tagged RBOHD (FLAG-RB-N) construct was transiently expressed in protoplasts derived from stable transgenic *Arabidopsis* Col-0 expressing *BIK1-HA* (BIK1) or *BIK1^C80A^* (BIK1^C80A^) for 16 hours and then either treated (+) or untreated (−) with 1 μM flg22 for 15 min. Total proteins (input) were subjected to immunoprecipitation with anti-HA agarose followed by immunoblot analysis using anti-FLAG. (**E**) Total photon counts were calculated in the given lines for 60 min following treatment with 200 nM flg22. Data are mean ± SE, *n* = 8 biologically intendent leaf discs and different letters indicate significant differences (one-way ANOVA; *P* < 0.05. (**F**) Determination of the titer of *Pst* DC3000 *hrcC^−^* in *Arabidopsis* plants of the indicated genotypes. Means ± SE, *n* = 6, different letters indicate significant differences at *P* < 0.05. (**G**) A working model of NO-potentiated BIK1-SNO in P/MAMP-triggered ROS production. P/MAMP recognition triggers the nitrosative burst, initiating the accumulation of NO, leading to the *S*-nitrosylation of BIK1 at Cys^80^ and subsequent BIK1 activation, including BIK1 phosphorylation and associated stabilization. The phosphorylation of BIK1 promotes its physical interaction with RBOHD to promote the oxidative burst and associated disease resistance.

To test the importance of *S*-nitrosylation in BIK1 activation, we mutated all the Cys residues in BIK1 replacing them with alanine (Ala) (BIK1^CA^). Subsequently, the extent of BIK1 phosphorylation in response to flg22 was determined. The absence of BIK1-SNO in BIK1^CA^ precluded BIK1 phosphorylation in response to either flg22 or GSNO (fig. S10), indicating the importance of BIK1-SNO for its phosphorylation during PTI. Thus, BIK1 Cys *S*-nitrosylation has a key function in BIK1 activation. Mass spectrometry was then undertaken in an attempt to identify the Cys residue(s) of BIK1 targeted for *S*-nitrosylation (table S1). Subsequently, an in vitro *S*-nitrosylation assay with individual Cys mutations suggested that one or more of four Cys residues might be a target for SNO formation (fig. S11). We next tested individual BIK1 Cys mutations for their potential ability to prevent pBIK1 formation in response to GSNO. Similar to wild-type BIK1, the BIK1^C4A^, BIK1^C140A^, and BIK1^C331A^ mutants exhibited phosphorylation in response to GSNO but not the BIK1^C80A^ mutant (fig. S12A), suggesting that precluding *S*-nitrosylation at Cys^80^ compromises the phosphorylation of BIK1 (pBIK1 formation). Thus, *S*-nitrosylation of BIK1 at Cys^80^ might be required for BIK1 phosphorylation and activation during PTI. On the basis of the crystal structure of BIK1 resolved by a recent study (*9*), Cys^80^ is located close to the adenosine triphosphate (ATP) docking site, and a root mean square deviation (RMSD) of atomic positions suggests BIK1 Cys^80^-SNO formation may trigger a conformation change of the ATP docking site resulting in enhanced ATP binding and also increased stability of BIK1 (Fig. 4A; figs. S12, B to D, and S13; and table S2). Computer modeling suggested that Cys^80A^ exhibits ATP binding activity (fig. S13, C to E), and this mutant remains functional because this mutant Cys^80A^ can rescue the *bik1* growth phenotype in *Arabidopsis* (fig. S12E) (*9*, *42*), but this mutation largely compromised its kinase activity (fig. S12F). Collectively, our data show that BIK1 Cys^80^-SNO formation might be important for PTI-triggered ROS production and plant immunity.

To test this posit, we therefore determined the biological consequence of a BIK1 mutation at Cys^80^, precluding *S*-nitrosylation, with respect to pBIK1 formation. The absence of BIK1 Cys^80^-SNO largely compromised pBIK1 formation in vivo in response to either flg22 or GSNO (Fig. 4, B and C). We also investigated the potential impact of BIK1^C80A^ on the flg22-triggered interaction between pBIK1 and RBOHD. Informatively, the absence of BIK1-SNO in the BIK1 ^C80A^ mutant reduced the interaction of BIK1 with RBOHD both in vitro (fig. S14, A and B) and in vivo (Fig. 4D). In aggregate, our data suggest that BIK1 Cys^80^-SNO formation might result in a conformational change that enhances ATP binding, thereby promoting pBIK1 formation in response to flg22, which subsequently drives the interaction of BIK1 with RBOHD.

Computer modeling suggested that Cys^80^ is located at the surface of the BIK1 interaction site with RBOHD, along with Lys^105^ and Lys^106^ (fig. S14C). Therefore, we tested whether *S*-nitrosylation of BIK1 at Cys^80^ might regulate the oxidative burst during PTI. The expression of wild-type *BIK1* in *bik1* plants completely restored ROS production upon flg22 treatment. Conversely, expression of *BIK1^C80A^* failed to reconstitute the oxidative burst to a similar level (Fig. 4E and fig. S15). Further, the absence of BIK1-SNO at Cys^80^ also reduced ROS production in response to elf18, and the application of GSNO did not further increase the level of ROS accumulation (fig. S16). Hence, *S*-nitrosylation of BIK1 at Cys^80^ may be required for an optimal oxidative burst following both flg22- and elf18-triggered PTI. Further, the stability of BIK1^C80A^ is not promoted by GSNO in vivo relative to wide-type BIK1 (fig. S17). Informatively, the expression of BIK1^C80A^ in *bik1* plants failed to restore resistance to *Pst* DC3000 *hrcC^−^*, as observed for expression of wild-type *BIK1* (Fig. 4F). Thus, *S*-nitrosylation of BIK1 at Cys^80^ promotes the oxidative burst and contributes to resistance against *Pst* DC3000 *hrcC^−^*.

## DISCUSSION

Our findings collectively establish a molecular framework for (S)NO function during PTI. Thus, following P/MAMP recognition, total cellular (S)NO, governed by AtGSNOR1, increases rapidly, leading to the *S*-nitrosylation of BIK1 at Cys^80^, promoting the phosphorylation of BIK1, thereby enhancing both the stability of BIK1 and its interaction with RBOHD. Together, this may help drive the oxidative burst leading to pathogen resistance. However, as (S)NO concentrations increase during the later stages of PTI, AtRBOHD may become *S*-nitrosylated at Cys ^890^, in a similar fashion to that which occurs during ETI (*25*), decreasing ROS synthesis. Analogously, our data show that high SNO levels decrease other PTI responses, including callose deposition and PTI-related gene expression. This complex NO-related molecular dialogue may therefore serve to both promote and subsequently curb PTI (Fig. 4G).

These data conceptually parallel the control of the plant transcriptional regulator, SRG1, whose transcription is rapidly activated by NO early in ETI, but subsequently, *S*-nitrosylation of SRG1 at Cys^87^ helps downgrade the associated immune response (*43*). A similar phenomenon occurs in mammals, where rapid, early *S*-nitrosylation of the surfactant protein D enables Toll-like receptor 4 dimerization and cognate activation promoting PTI (*44*, *45*). However, as NO levels increase over time, both the p50 and p65 subunits of the immune activator, nuclear factor κB, become *S*-nitrosylated, inhibiting immune responses (*46*, *47*). Our findings suggest similar complex roles for NO-based redox regulation of plant PTI, where future insights may provide improved strategies for the development of disease resistance.

## MATERIALS AND METHODS

### Plant materials and growth conditions

*Arabidopsis* accession Columbia (Col-0) was used in this study as wild-type, and *Arabidopsis* T-DNA insertion lines *bak1-4* (SALK_116202) and *fls2* (SAIL_691C4) were obtained from the Nottingham Arabidopsis Stock Centre. The mutant lines *bik1*, *gsnor1-3*, *par2-1*, and *nox1* have been described previously (*10*, *31*, *33*, *34*). Transgenic *BIK1-HA* and *BIK1^C80A^* driven by its native promoter in *bik1* background and *BIK1-HA*, *BIK1^C4A^-HA*, and *BIK1^C80A^*-HA driven by 35*S* promoter in Col-0 background were generated in this study.

*Arabidopsis* seeds were surface-sterilized and planted on ½ MS (Murashige and Skoog basal) agar medium. For NO-mediated *Arabidopsis* hypocotyl length assay, seeds were geminated on ½ MS, containing 0.8% sucrose and 0.8% agar at pH 5.6 for 5 days with or without indicated concentration of SNP. The 10-day-old Col-0 and transgenic seedlings grown on ½MS agar medium were used for treatment with chemicals or pathogens.

*Arabidopsis* plants were grown in soil under conditions 21 to 23°C, a 12-hour light/12-hour dark photoperiod for 4 weeks for pathogen inoculation assay, protoplasts isolate and transient expression assay, ROS burst assay, and callose deposition assay. *Arabidopsis* plants were grown in soil under conditions 21 to 23°C, a 16-hour light/8-hour dark photoperiod for genetic transformation and seed collection. The transgenic plants were selected in ½ MS agar medium with kanamycin (50 μg ml^−1^), and homozygous lines with a single insertion were used for experiments.

### Plasmid constructs and protoplast transient assays

The coding region of *BIK1* was amplified from Col-0 cDNA and then cloned to pDONR207 to obtain *pDONR207-BIK1* by Gateway BP reaction (Invitrogen, UK), and the *BIK1* point mutations *BIK1^C80A^* and *BIK1^C4A^* were constructed by sequentially mutating in *pDONR207-BIK1* to generate *pDONR207- BIK1^C80A^* and *pDONR207- BIK1^C4A^* using the Quick Change II XL Site-Directed Mutagenesis Kit (200521, Agilent). *pDONR207-BIK1*, *pDONR207- BIK1^C80A^*, and *pDONR207- BIK1^C4A^* were then subcloned into PGWB14 (*48*) by LR reaction (Invitrogen, UK) to generate HA-tagged vector *BIK1-HA*, *BIK1^C80A^-HA*, and *BIK1^C4A^-HA* driven by 35*S* promoter, respectively. These resulting plasmids were transformed into wild-type Col-0, respectively, by the floral dip method (*49*). The 1260-bp BIK1 promoter was fused to coding sequence of *BIK1* or *BIK1^C80A^* and then subcloned into PGWB13 vector (*48*) by Gateway cloning technology (Invitrogen) to generate HA-tagged *BIK1-HA* or *BIK1^C80A^-HA*, respectively. These resulting plasmids were transformed into *bik1* background, respectively, by the floral dip method (*49*).

The 1- to 1128-bp fragment of *RBOHD* (*RBOHD-N*) was amplified from construct *RBOHD-FLAG* (*10*) and then cloned into pDONR207 to generate entry clone *pDONR207-RBOHD-N*. The *pDONR207-RBOHD-N* was then subcloned into vector pEarlygate202 to obtain FLAG-tagged vector *FLAG*-*RBOHD-N. pDONR207-BIK1* was subcloned to pDEST-HisMBP or pDEST17) to generate 6xHIS-tagged or maltose binding protein (MBP)–tagged BIK1 recombinant protein expression vector *pDEST-HisMBP-BIK1* or *pDEST17-BIK1*, respectively. The *pDONR207-RBOHD-N* was subcloned into pDEST15 to generate glutathione *S*-transferase (GST)–tagged *RBOHD-N* recombinant protein expression vector *pDEST-15-RBOHD-N*.

For the protoplast transfection procedure, 0.1 ml of *Arabidopsis* protoplasts derived from 4-week-old Col-0, *par2-1*, or *gsnor1-3* mutant under short days, at a density of 2 × 10^5^ per ml, was transfected with 5 μg of plasmid DNA as described method (*50*).

### Pathogen inoculation, callose deposition, and NO detection

Bacteria strains *P. syringae* pv. *tomato* (*Pst*) DC3000 and *Pst* DC3000 *hrcC^−^* (*HrcC^−^)* mutant were cultured overnight at 28°C in king’s B medium with rifampicin (25 μg/ml), and the bacteria were collected by centrifugation at 3500*g* for 3 min. The bacteria *Pst* DC3000 were washed two times with 10 mM MgCl_2_ and resuspended in 10 mM MgCl_2_ for inoculation. Pathogen inoculations and bacterial growth were performed as described (*51*). Briefly, leaves from 4-week-old plants were syringe-inoculated with *Pst* DC3000 with a density of 10^6^ colony-forming units (CFU)/ml, and leaves were spray inoculated with *Pst* DC3000 *hrcC^−^* at a density of 5 × 10^8^ CFU/ml supplemented with silwet L-77 (0.02%). To measure bacterial growth in planta, two 5 mm in diameter leaf discs from each plant, with a total of six leaf discs being pooled together representing one biological replicate with three biological replicates for bacterial growth assays. For the flg22 protection assay, 4-week-old plants were firstly infiltrated with 1 μM flg22 or H_2_O 24 hours before infiltration of *Pst* DC3000 and then measured bacterial growth.

For the detection of callose deposition, 4-week-old *Arabidopsis* leaves were infiltrated with 1 μM flg22 using a needleless syringe for 24 hours, and then callose deposition was performed as previously described (*52*).

In situ NO detection was performed as described (*25*) with NO-sensitive dye DAF-FM DA (ab145295, Abcam). The stock solutions were prepared in dimethyl sulfoxide with 5 mg/ml and diluted to working concentrations with 5 μg/ml in phosphate-buffered saline (PBS) buffer. Three-day-old *Arabidopsis* seedlings were inoculated with 1 μM flg22 in combination with or without cPTIO and then subjected to DAF-FM DA staining for 40 min. After washing three times with PBS buffer, the fluorescence of *Arabidopsis* roots was monitored under a Leica SP5 laser confocal microscope. Each individual root is recognized as one biological replicate.

### ROS burst assay

The PAMP-triggered ROS burst was conducted as previously described. Briefly, *Arabidopsis* leaf discs were harvested with a cork borer from 4-week-old plants grown in short day conditions and placed in opaque white costar flat-bottom microplate with 100 μl water per well. The plate was placed in a growth room with cover overnight, and ROS production was measured using a luminol-dependent assay by replacing water with 100 μl of 200 nM flg22 (RP19986, GeneScript, USA) or 200 nM elf18 (crb1000748, Cambridge Research Biochemicals) and 30 μM L-012 (*40*), supplemented with the indicated concentration of chemicals. Luminescence was measured immediately using a TECAN Infinite 200 PRO microplate reader, and the ROS production was indicated as means of relative light units. Each individual leaf disc from one plant was recognized as one biological replicate for the ROS burst assays with at least six biological replicates.

### Recombinant protein purification and in vitro kinase assays

*Escherichia coli* strain BL21(*DE3*) was used in this study for all recombinant protein expression and purification. BIK1 fusion protein expression was induced by adding 0.4 mM IPTG (isopropyl-β-d-1-thiogalactopyranoside) to a 150-ml culture at optical density at 600 nm (OD_600_) = 0.5 for additional 6 hours at 28°C. cells were harvested, and the recombinant proteins were purified according to the manufacturer’s instructions using amylose magnetic beads (E8035S, New England Biolabs, USA) for MBP-tagged and Ni-NTA resins (88221, Thermo Fisher Scientific) for HIS-tagged recombinant protein purification. GST-tagged RBOHD N terminus (*RBOHD-N*) expression was induced by addition 0.1 mM IPTG to a 200-ml culture at OD_600_ = 0.6 for additional 18 hours at 16°C, and GST fusion proteins were purified according to the manufacturer’s instructions using Glutathione Sepharose 4B (GE Healthcare).

In vitro kinase assays were performed as previously described (*11*, *40*) with modification. All purified recombinant protein was equilibrated with 1× HEN buffer [250 mM Hepes (pH7.7), 1 mM EDTA, and 0.1 mM neocuproine] before treating with different concentration of NO donor for 30 min at 24°C under darkness. Excess NO donor was removed by Zeba Spin Desalting Columns (7K, Thermo Fisher Scientific), and the resulting proteins were used for in vitro kinase assay in 20-μl volumes of reaction buffer [20 mM tris-HCl (pH 7.5), 10 mM MgCl_2_, 1 mM CaCl_2_, and 200 mM ATP] for 30 min at 30°C under dark. The reaction was stopped by adding 5 μl of 5× Leammli sample buffer, and the resulting samples were subjected to immunoblot against anti-phospho-(Ser/Thr) Phe antibody (ab17464, Abcam) followed by anti–rabbit–horseradish peroxidase (HRP) secondary antibody (ab205718, Abcam).

### In vitro pull-down assays

Recombinant proteins were purified separately, and the BIK1 recombinant protein was pretreated with 10 μM NO donor GSNO or 100 μM DTT for 30 min at 24°C under darkness. Excess NO donor was removed by Zeba Spin Desalting Column, and the resulting proteins were used for in vitro GST pull-down. GST pull-down was performed by incubating GST-RBOHD-N (GST-RB-N) with MBP or MBP-BIK1 in a 400-μl pull-down buffer [30 mM Hepes, 100 mM NaCl, and 0.2% Triton X-100 (pH 7.5)] with Glutathione Sepharose 4B resin. The pull-down reactions were incubated at 4°C for 4 hours under dark with gentle shaking, and lastly, the Sepharose 4B beads were centrifuged 500*g* for 5 min and washed for five times with washing buffer [50 mM tris-Cl (pH 8.0), 150 mM NaCl, and 0.1 mM EDTA]. Protein bound to Sepharose 4B resin was eluted by 1× Leammli sample buffer and then subjected to immune blotting using anti-MBP antibody (E8032S, NEB) and anti-mouse-HRP secondary antibody (7076, Cell Signaling Technologies).

### Yeast two-hybrid assay (Y2H)

The yeast strain Y187 was used for yeast two-hybrid assay to test the interaction between BIK1 and N-RBOHG. Briefly, the full-length BIK1 fused with the pGADT7 vector harboring an activation domain (AD), and RBOHD-N terminus fused with DNA binding domain of GAL4 in pGBKT7 (BD-RBOHD). The empty vector pGADT7 (AD) and pGBKT7 (BD) were used as a negative control, and the transformation was plated on SD-Leu-Trp or SD-Leu-Trp with X-α-Gal in the presence of indicated SNP.

### *S*-nitrosylation assays

The BST was performed to detect protein *S*-nitrosylation in vivo and in vitro according to that previously described (*30*) with minor modifications. For in vitro assays, the purified recombinant protein was equilibrated with 1× HEN buffer before treating with the stated concentration of the given NO donor in 100-μl volumes for 30 min in darkness at room temperature. Excess NO donor was removed by Zeba Spin Desalting Column followed by blocking free thiols with 300 μl of blocking buffer (1× HEN buffer with 2.5% SDS and 20 mM *N*-ethylmaleimide) for 30 min at 50°C. The blocking buffer was removed by adding 800 μl of cold acetone to precipitate proteins for 30 min in −20°C and then centrifuged at 15,000*g* for 20 min at 4°C. Precipitated proteins were washed twice with 70% cold acetone, and the resulting proteins was resuspended in 50 μl of labeling buffer (1× HEN buffer with 5 mM sodium ascorbate and 1 mM biotin-HPDP) for 2 hours in room temperature under dark. Where indicated, −Asc refers to the omission of the ascorbate step in the labeling buffer was used as a negative control. The samples were denatured by adding 50 μl of 2× Leammli sample buffer without DTT, unless otherwise indicated, and the samples were subjected to immune blotting against an antibiotin HRP-linked antibody (7075, Cell Signaling Technologies).

For in vivo assays, protein total *S*-nitrosylation was performed by inoculating 10-old-day *Arabidopsis* wild-type seedlings with 1 μM flg22 or 10 μM GSNO for 15 min and then subjected to BSTs with an antibiotin HRP-linked antibody (7075, Cell Signaling Technologies). The labeling buffer without sodium ascorbate was used as a negative control. Analysis of BIK1-SNO in vivo was conducted using BST followed by immune blot against anti-HA antibody (ab18181, Abcam) and anti–mouse-HRP secondary antibody (7076, Cell Signaling Technologies).

### Immunoprecipitation assays

To examine the effects of NO on the association between BIK1 and RBOHD-N, the *Arabidopsis* protoplasts derived from 4-week-old *BIK1-HA* expressing lines under short day condition were transfected with FLAG-tagged construct *FLAG*-*RBOHD-N* as described (*50*). After 16 hours of incubation, protoplasts were treated with 1 μM flg22 for 15 min in combination with 10 μM GSNO or 200 μM cPTIO as indicated, and the total protein was isolated with an extraction buffer [50 mM tris-HCl (pH 7.5), 50 mM NaCl, 10% glycerol, and complete protease inhibitor]. Total protein was incubated with an anti-HA agarose (Thermo Fisher Scientific) for 4 hours followed by washing for five times. The immunoprecipitates were subjected to immune blot using anti-FLAG antibody (F3165, Sigma-Aldrich).

### Structural modeling

The structure of BIK1 was obtained from the UniProt (https://uniprot.org/) (predicted in AlphaFold2), similar to its crystal structures Protein Data Bank code 5TOS (RMSD: 2.17 Å). In contrast, the ─SH group of Cys^80^ is modified to the ─SNO group in the BIK1 structure in the Maestrothe Schrodinger suite. The proteins models (BIK1-C80 and BIK1-C80-SNO) were prepared with the Protein Preparation Wizard included in Maestro under the OPLS_2005 force field: Hydrogen atoms were added to the repaired structures at physiological pH (7.0) with the PROPKA tool to optimize the hydrogen bond network, all water molecules were removed, C- and N-terminal cappings were added, disulfide bonds were assigned, and constrained energy minimizations were carried out on the full-atomic models until the RMSD of the heavy atoms converged to 0.3 Å. The ligand (ATP) was downloaded from PubChem Database (https://pubchem.ncbi.nlm.nih.gov/).The LigPrep tool in the Schrodinger suite was introduced for geometric optimization by using the OPLS_2005 force field. Ionization states of ligands were calculated with the Epik tool using Hammett and Taft methods in conjunction with ionization and tautomerization tools. Then, glide (*53*) performed the docking procedure in the Schrodinger suite software. The best-scored pose for ATP was chosen as the initial structure for molecular dynamics (MD) simulations. To identity the effect of mutation on structure or the ATP biding, Desmond (*54*) was used to execute MD simulations on the BIK1-C80 and BIK1-C80-SNO structures and their complexes with ATP, respectively. TIP3P model was used to build the system in the orthorhombic periodic boundary conditions at the distances of 10-Å units. Moreover, Na^+^ and Cl^−^ were added to neutralize the charge of all systems and bring salt concentration to 150 mM. The simulation was processed under the isothermal-isobaric ensemble (NPT) environment with constant number of molecules, constant temperature, and pressure conditions at 300 K and 1.013 bar. The entire system including BIK1 proteins, ligands, ions, and solvents is minimized by a mongrel method of steep descent and limited-memory Broyden-Fletcher-Goldfarb-Shanno algorithm iterated up to 5000 times. The MD simulation lasted for 200 ns with time step of 0.1 ps. In addition, RMSD, root mean square fluctuation, and ligand-protein interaction were used to evaluate the stability and deviation of protein and small molecule from initial state of the interacting complexes. Final output results were visualized in PyMOL.

### Molecular mechanics/generalized Born surface area

The molecular mechanics/generalized born surface area (MM/GBSA) of the Prime module (*55*) was applied to calculate the binding free energy of ligand-protein complex trajectories via the following equations (*56*)

$$\Delta G_{\text{bind}}=\Delta E_{\text{MM}}+\Delta G_{\text{sol}}$$ (1)

$$\Delta E_{\text{MM}}=\Delta E_{\text{vdW}}+\Delta E_{\text{Coulomb}}+\Delta G_{\text{Covalent}}$$ (2)

$$\Delta G_{\text{sol}}=\Delta G_{\text{GB}}+\Delta G_{\text{Lipo}}+\Delta G_{\text{Correction}}$$ (3)

$$\Delta E_{\text{Correction}}=\Delta E_{\text{Hbond}}+\Delta E_{\text{Packing}}+\Delta E_{\text{SelfCont}}$$ (4)

The stable last-frame snapshot extracted from the MD simulation is estimated by the Prime model to evaluate the binding free energy performance.

### Gene expression analysis

To determine the relative gene expression level, total RNA was extracted from 100-mg plant tissue using an RNA isolation kit (Agilent Technologies), and first-strand cDNA was synthesized via reverse transcription reaction with the Revert Aid First Strand cDNA Synthesis Kit (K1622, Thermo Fisher Scientific). Gene expression was analyzed by quantitative reverse transcription polymerase chain reaction using the LightCycler 480 Real-Time PCR System (Roche) with *UBQ10* as an internal control, and gene-specific primers were shown in table S3.

### Statistical analysis

Data were presented as mean ± SE. One-way analysis of variance (ANOVA) followed by Tukey’s test or two-tailed *t* test was used for the statistical analysis. *P* ≤ 0.05 was considered as significant.
