# Supplementary material for: S-nitrosylation of a receptor-like cytoplasmic kinase regulates plant immunity
Source: Sci Adv. 2024 Mar 15;10(11):eadk3126. doi: 10.1126/sciadv.adk3126 (PMC10942119; doi:10.1126/sciadv.adk3126)
Supplement: Supplementary file 1 — Figs. S1 to S17 Tables S1 to S3 [file sciadv.adk3126_sm.pdf]

Science Advances
AAAS

Supplementary Materials for

S-nitrosylation of a receptor-like cytoplasmic kinase regulates plant immunity

Beimi Cui *et al.*

Corresponding author: Fengquan Liu, fqliu20011@sina.com; Gary J. Loake, gloake@ed.ac.uk

Sci. Adv. **10**, eadk3126 (2024)
DOI: 10.1126/sciadv.adk3126

This PDF file includes:

Figs. S1 to S17
Tables S1 to S3

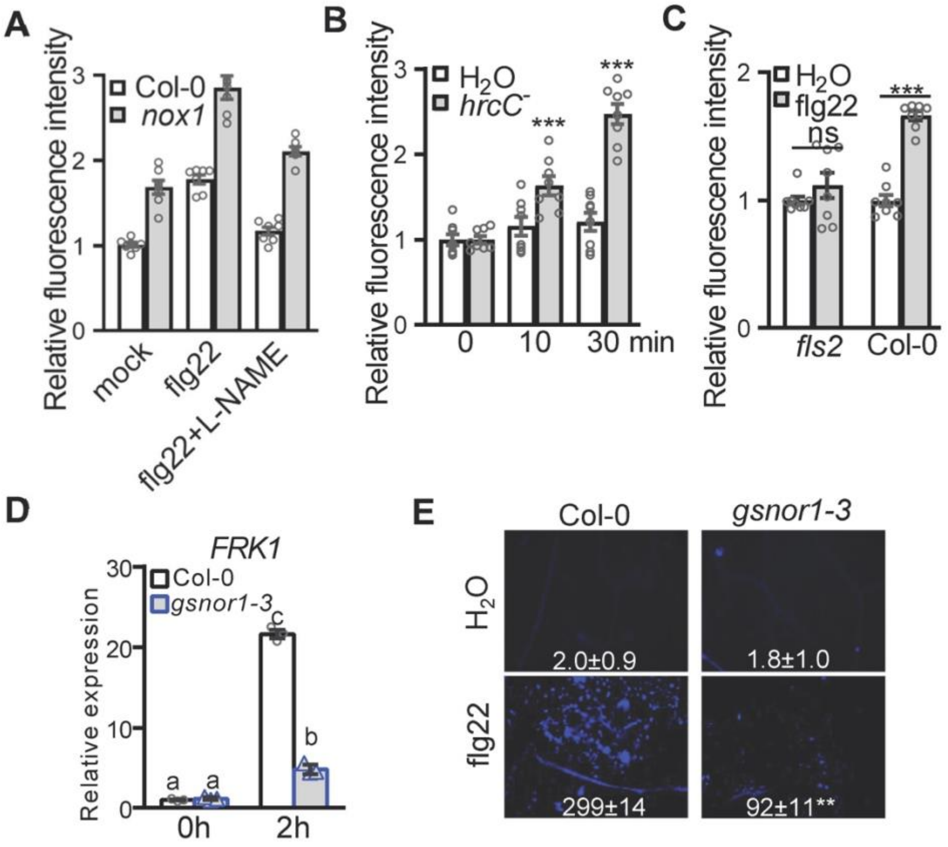

Fig. S1 Nitric oxide functions in PTI.

(**A-B**) NO levels were determined in Col-0 and *nox1* mutant following 1 μM flg22 with or without L-NAME for 10 mins (A), or in Col-0 following challenged with *Pst* DC3000 *hrcC*$^-$ (*hrcC*$^-$) for either 0, 10 or 30 mins (B) by DAF-FM DA staining, a marker for NO accumulation. Roots of *Arabidopsis* seedlings were challenge with indicated flg22 or pathogen followed by quantification of DAF-FM DA staining to determine NO accumulation. Asterisks indicate statistically significant differences compared to H_2O treatment at P<0.05, mean ± SE, n = 7. (**C**) Roots of wild-type and *fls2 Arabidopsis* seedlings treated with 1 μM flg22 for 10 mins followed by quantification of DAF-FM DA staining. Asterisks indicate statistically significant differences compared to H_2O treatment at P<0.05, mean ± SE, n = 7. (**D**) Flg22-induced *FRK1* gene expression was measured by RT-qPCR. The relative expression levels were normalized to *UBQ10* and values shown are the mean ± SE, *n* = 3 and different letters indicate significant differences (one-way ANOVA; P < 0.05). (**E**) Callose accumulation in the indicated plant lines following treatment with flg22 for 24 hours as determined by aniline blue staining and subsequently quantification. Average callose accumulation per cm^2 is presented for each genotype (means ± SE, n=6). ******, P < 0.01 by two-tailed *t*-test.

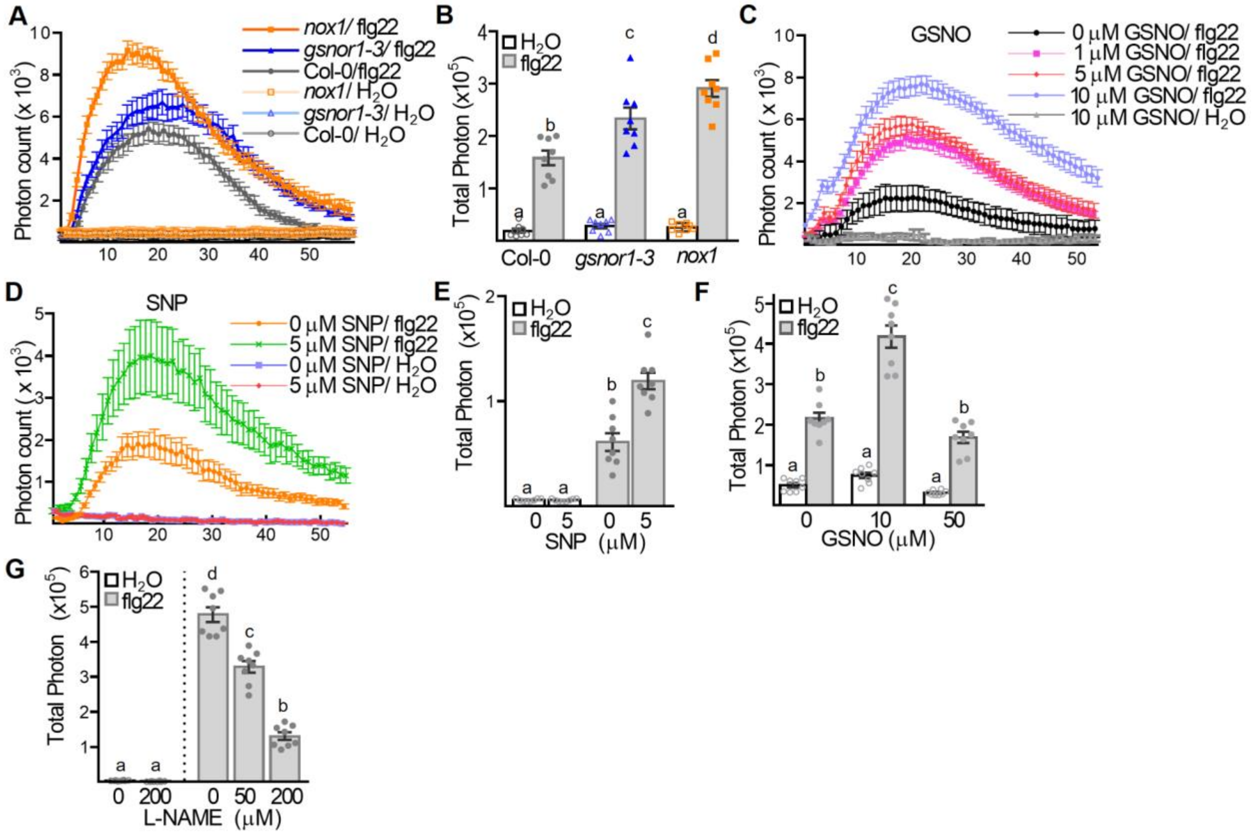

Fig. S2 Nitric oxide potentiates the flg22-induced ROS burst.

(**A)** ROS burst over 60 mins determined in the given genotypes in response to the stated cues. (**B**) Total ROS determined in wild-type Col-0, *gsnor1-3*, *nox1* plants in response to 200 nM flg22. (**C-G**) Flg22-triggered ROS burst was detected in wild-type Col-0 plants treated with the given chemicals. The time course (**C** and **D**) and total of photon counts (**E-G**) were calculated. Values are mean ± SE, n=8 of leaf disks as biologically independent samples. Different letters indicate significant differences at $P < 0.05$. All experiments in this figure were repeated three times with similar results.

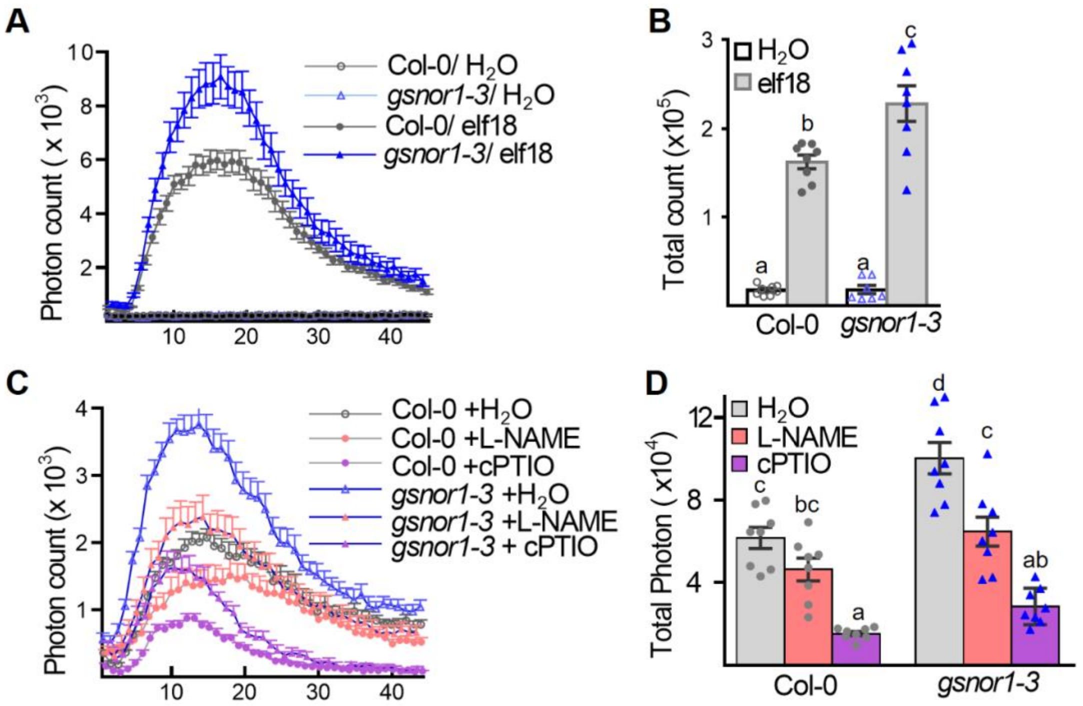

Fig. S3 Nitric oxide potentiates the elf18-induced ROS burst.

(**A**) Time-course of ROS production in response to 200 nM elf18 treatment in wild-type Col-0 and *gsnor1-3* plants. (**B**) Total photon counts calculated from (A). (**C and D**) Elf18-triggered ROS burst was detected in Col-0 and *gsnor1-3* plants treated either with or without L-NAME / cPTIO. Leaf discs were maintained under darkness overnight with H_2O. Subsequently, the H_2O was replaced with 200 nM elf18 either with or without 200 μM L-NAME / 200 μM cPTIO as indicated, followed by luminescence measurement. Values are mean ± SE, n=8, different letters indicate significant differences at $P < 0.05$. All experiments in this figure were repeated three times with similar results.

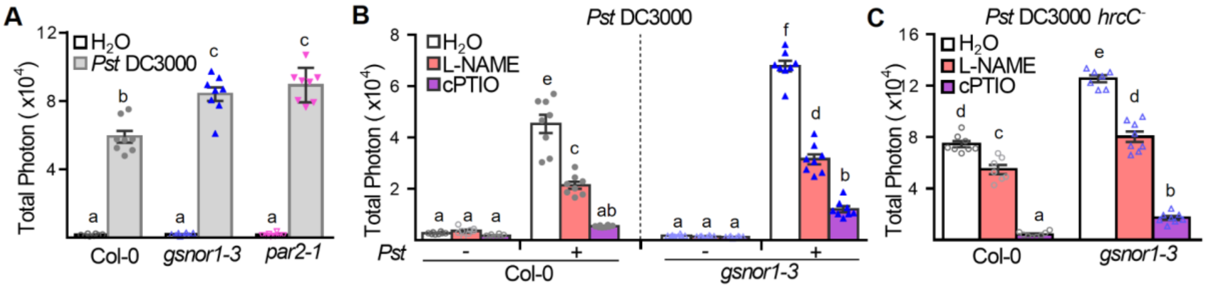

Fig. S4 Nitric oxide potentiates the *Pst* DC3000-induced ROS burst.

Total photon counts of luminescence assay reporting the ROS burst in response to *Pst* DC3000 (**A**) or *Pst* DC3000 (*Pst*) either with or without L-NAME / cPTIO (**B**) or *Pst* DC3000 *hrcC*$^{-}$ either with or without L-NAME/cPTIO (**C**). The values represent mean ± SE, n=8, different letters indicate significant differences at $P < 0.05$.

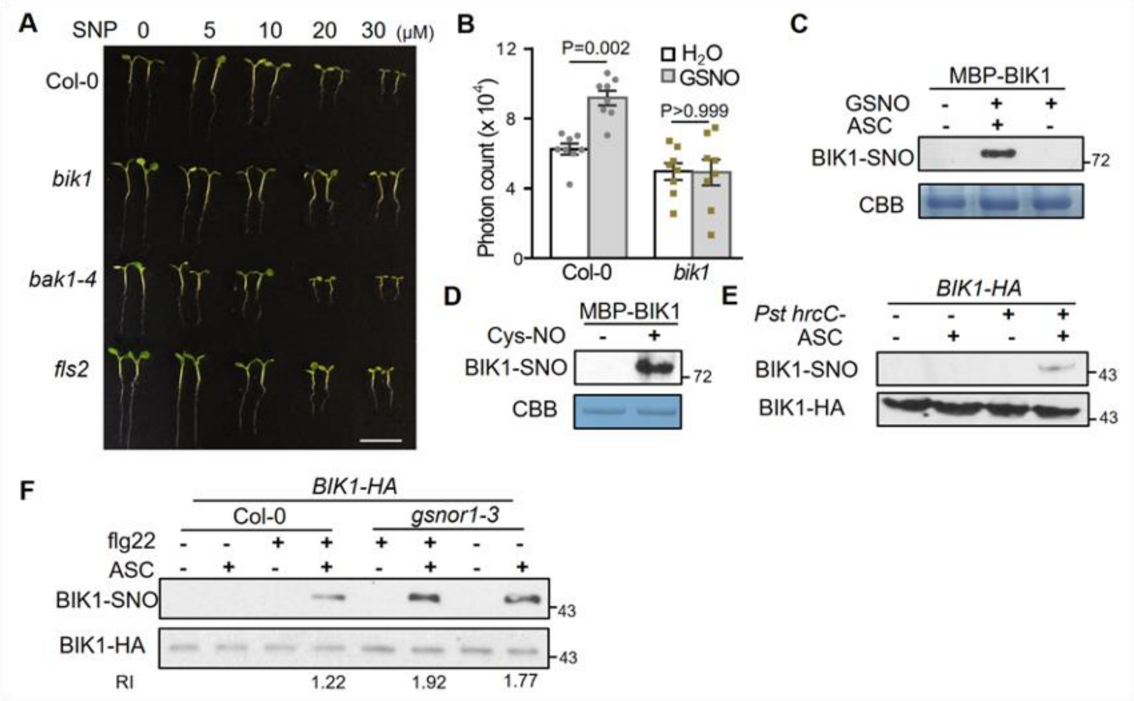

Fig. S5 BIK1 is *S*-nitrosylated during PTI.

(**A**) Phenotype of *Arabidopsis* seedlings grown on medium with the NO donor, SNP. Seedlings of wild-type Col-0, *bik1*, *bak1-4* and *fls2* were grown on ½ MS media supplied with the indicated concentrations of SNP for 5 days. Scale bar, 1 cm. (**B**) Total photon counts were calculated in wild-type Col-0 and *bik1* plants following treatment with 200 nM elf18 together with either H_2O or 2 µM GSNO. Data are mean± SE, n= 8 biologically intendent leaf discs and *P* values as shown are one-way ANOVA analysis. (**C-D**) BIK1-SNO formation detection *in vitro*. Recombinant maltose binding protein (MBP)-BIK1 was subjected to the biotin-switch assay (BSA) following treatment with the NO donor 100 µM GSNO (C) or 100 µM Cys-NO (D). Protein loading was visualized by coomassie brilliant blue (CBB) staining. (**E-F**) PAMP-induced BIK-SNO formation. Total protein extract from *Pst* DC3000 *hrcC*$^-$ (E) or flg22 (F) challenged *BIK1-HA* expressing plants for 15 mins was subjected to the BSA. Ascorbate (ASC) was used as a control for SNO formation. Lower panel indicates total BIK1-HA protein for each sample detected with an anti-HA antibody.

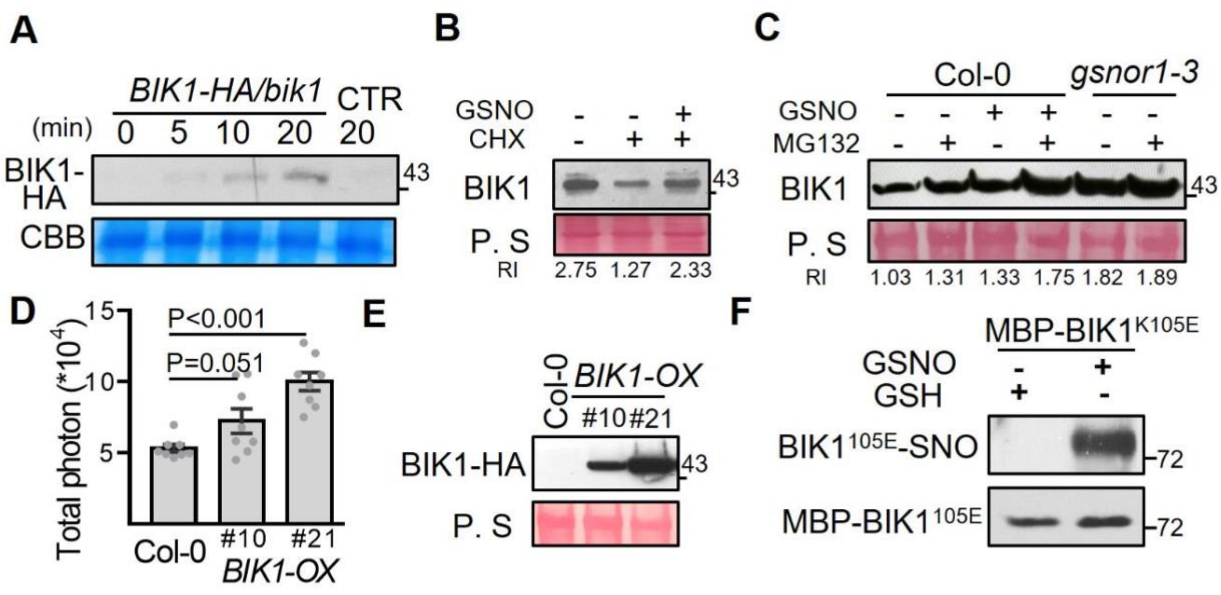

Fig. S6 Nitric oxide increases BIK1 stability and PAMP-triggered BIK1 phosphorylation.

(**A**) BIK1-HA abundance was detected after sodium nitroprusside (SNP) treatment. Total protein extracted from 10 μM SNP treated *BIK1-HA* expressing lines was subjected to a western blot assay utilizing a HA antibody after treatment with SNP. Coomassie brilliant blue (CBB) staining served as a loading control. (**B**) *BIK1-HA* expressing plants in a Col-0 background were treated with 100 μM cycloheximide (CHX) and 10 μM GSNO for 8 hours before total protein was examined with immunoblotting. Ponceau S (P.S) staining indicates protein loading. (**C**) BIK1 levels determined in wild-type Col-0 and *gsnor1-3* plants. Col-0 or *gsnor1-3* plants were treated with either 10 μM GSNO or 120 μM MG132 for 8 hours. Subsequently, total protein was extracted for detection of BIK1 levels utilizing an anti-BIK1 antibody. (**D-E**) Total ROS production (D) from transgenic lines expressing different lev els of BIK1 (E) was calculated in the presence of 200 nM flg22 for 60 mins. Values in (D) are mean ± SE, n=8. one-way ANOVA. Western blot in E shows protein levels determined with a HA-antibody. P.S indicates protein loading levels. (F) The phopho-mutant version BIK1^{K105E} was *S*-nitrosylated. Recombinant MBP-BIK1 ^{K105E} was subjected to the biotin-switch assay (BSA) following treatment with the NO donor GSNO (10 μM) or with 10 μM glutathione (GSH).

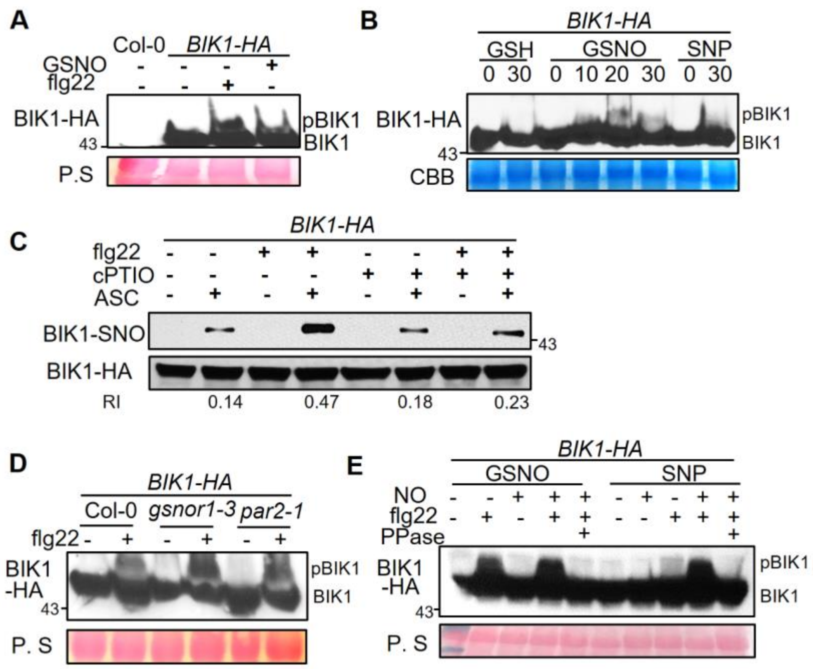

Fig. S7 flg22 induced BIK1-SNO formation

(**A-B**) BIK1 phosphorylation (pBIK1) was detected in *BIK1-HA* expressing lines treated with the given chemicals. Wild-type Col-0 or *BIK1-HA* expressing plants were treated with either 10 µM GSNO or 1 µM flg22 for 15 mins (A) or with the indicated concentrations of glutathione (GSH), GSNO, or SNP for 0, 10, 30 mins (B) prior to a mobility-shift assay. (**C**) 10-day-old seedlings of *BIK1-HA* expressing plants were treated with 1 µM flg22 either with or without 200 µM cPTIO for 15 mins and then total protein extracts were subjected to the biotin-switch assay (BSA). Ascorbate was used as control for SNO formation. Lower panel indicates total BIK1-HA for each sample detected by employing an anti-HA antibody. Relative intensity (RI) indicates the relative intensity of BIK1-SNO compared to BIK1-HA determined by image J. (**D**) *BIK1-HA* was transiently expressed in protoplasts derived from either wild-type Col-0, *gsnor1-3* or *par2-1* plants and subsequently treated with 1 µM flg22 for 15 mins before mobility-shift assay analysis. (**E**) *BIK1-HA* expressing plants were treated with either 10 µM GSNO, 200 nM flg22 or 20 µM SNP for 15 mins and subsequently the total protein extract was either treated with λ protein phosphatase (PPase) (+) or remained untreated (-) before mobility-shift analysis. Coomassie brilliant blue (CBB) or Ponceau S (P. S) staining served as a loading control.

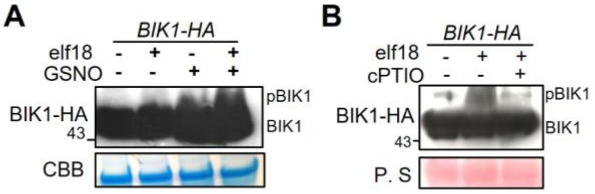

Fig. S8 Nitric oxide potentiates elf18-induced BIK1 phosphorylation.

(**A**) BIK1 phosphorylation (pBIK1) formation in *BIK1-HA* expressing *Arabidopsis* protoplasts treated with 1 μM elf18 and 10 μM GSNO either individually or in combination and subsequently analyzed by mobility-shift assay using an anti-HA immunoblot. CBB staining served as a loading control. (**B**) pBIK1 formation in *BIK1-HA* expressing *Arabidopsis* protoplasts treated with 1 μM elf18 and 10 μM GSNO either individually or in combination for 15 mins and subsequently analysed by mobility-shift assay using an anti-HA immunoblot. P. S staining served as a loading control.

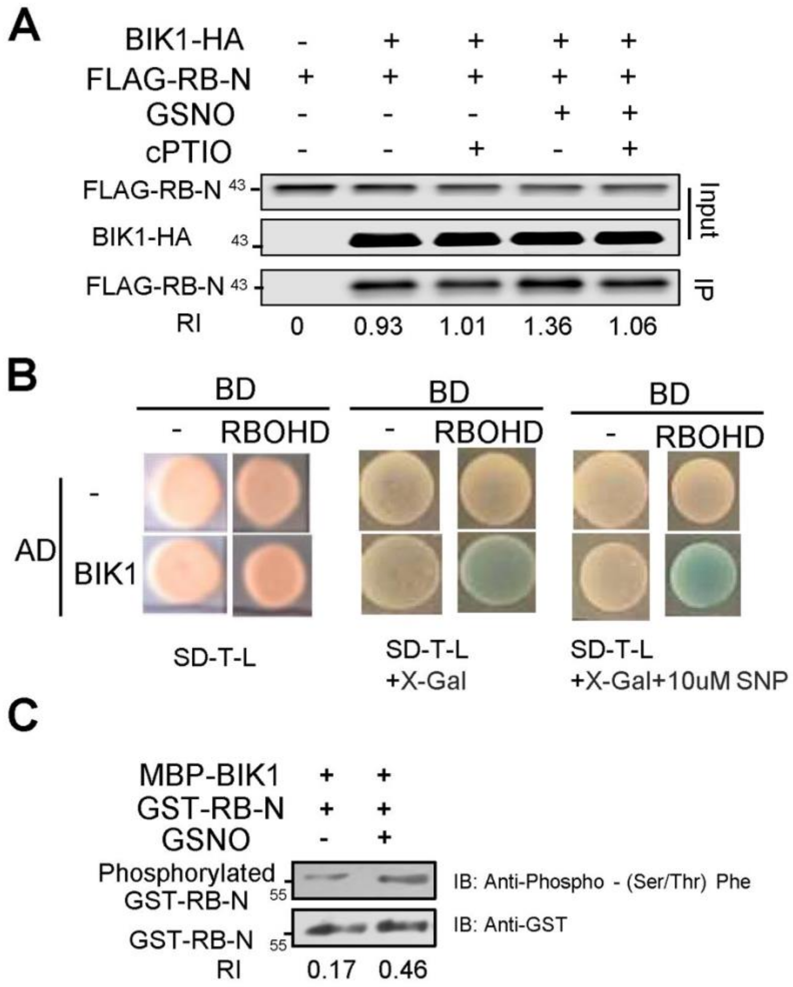

Fig. S9 Nitric oxide potentiates BIK1-dependent RBOHD phosphorylation. (A) Co-IP assays of BIK1 and RBOHD-N. Flag-tagged RBOHD (FLAG-RB-N) construct was transiently expressed in protoplasts derived from stable transgenic *Arabidopsis* Col-0 expressing BIK1-HA for 16 hours and then treated (+) or untreated (-) with 10 μM GSNO, or 200 μM cPTIO for 15 mins. Total proteins (input) were subjected to immunoprecipitation with anti-HA agarose followed by immunoblot analysis employing anti-FLAG. All experiments were performed three times with similar results. **(B)** NO donor SNP enhances the interaction between BIK and RBOHD in yeast. The yeast cells of strain Y187 expressing the indicated plasmid combinations were grown on either SD-LT media or SD-LT media plus X-α-Gal. **(C)** MBP-BIK1 recombinant protein either pretreated with GSNO or unpretreated was incubated with GST-RBOHD-N-terminus (GST-RB-N) recombinant protein for 30 mins followed by an *in vitro* kinase assay. Subsequently, an immune blot assay using an anti-phospho antibody was undertaken. An anti-GST antibody served as loading control.

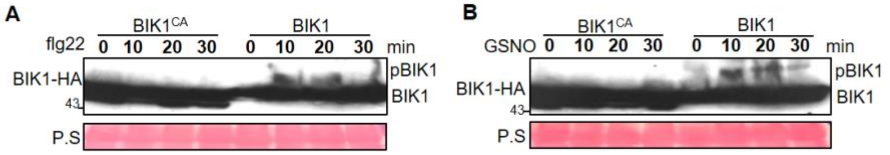

Fig. S10 BIK1 cysteine residues are required for flg22- and GSNO-promoted pBIK1 formation.

(A-B) 10-day-old seedlings expressing *BIK1-HA* (BIK1) and *BIK1-HA*^{CA} (BIK1^{CA}) were treated with 1 μM flg22 (A) or 10 μM GSNO (B) for the indicated times. Subsequently total protein extract was subjected to the mobility-shift assay using an anti-HA immunoblot. Ponceau S (P. S) staining served as loading control.

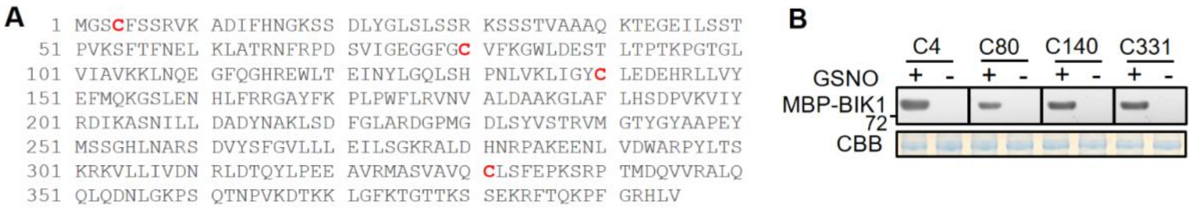

Fig. S11 Identification of the site of BIK1 *S*-nitrosylation.

(**A**) Amino acid sequence of BIK1. The cysteine (C) residues in BIK1 are highlighted in red. (**B**) Biotin switch assay detecting BIK1-SNO formation with the indicated BIK1 C mutations. C4: C80A/C140A/C331A. C80: C4A/C140A/C331A. C140: C4A/C80A/C331A. C331: C4A/C80A/C140A.

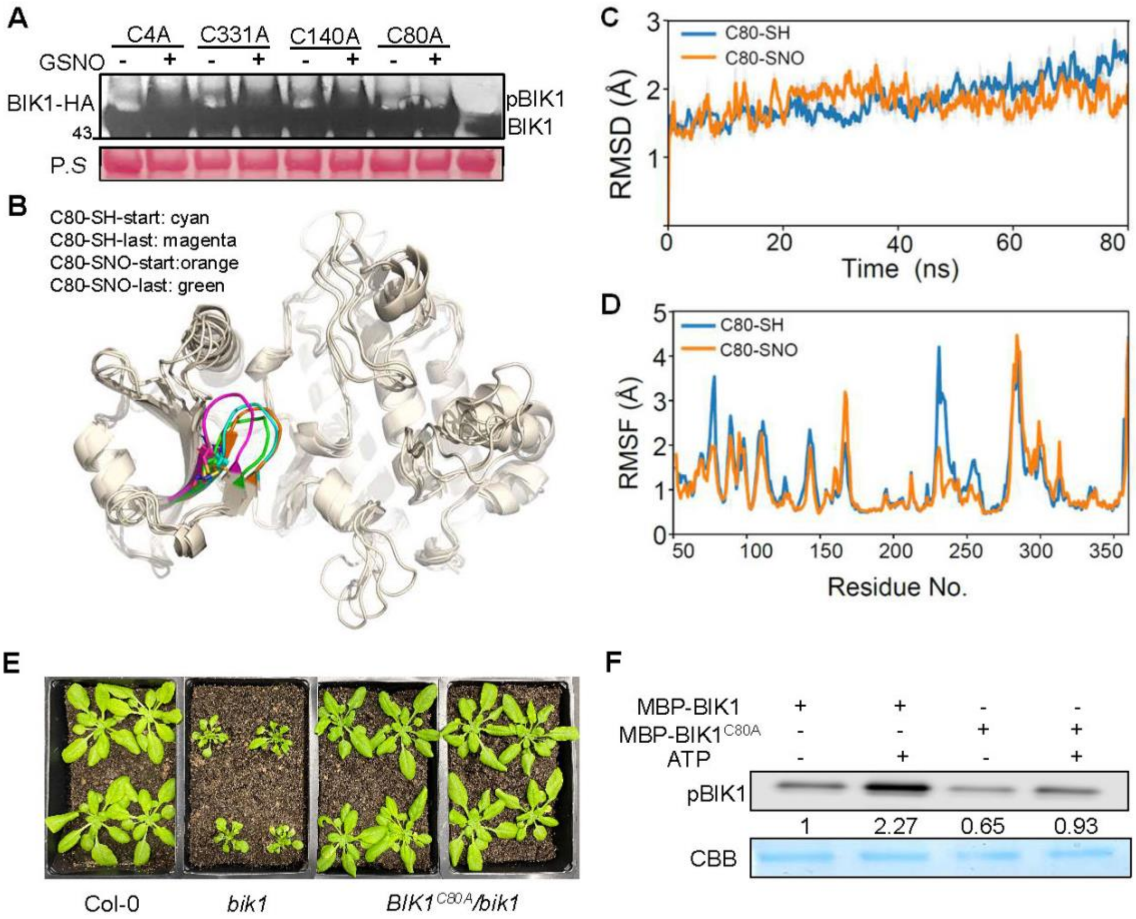

Fig. S12 *S*-nitrosylation of BIK1 at Cys^{80} promotes BIK1 phosphorylation and stability.

(A) BIK1-Cys^{80} is required for GSNO-induced BIK1 phosphorylation. Total protein extract from indicated protein expressing line treated with 10 μM GSNO was subjected to the mobility-shift assay using anti-HA immunoblot. (**B**) Molecular modeling to show the BIK1 ATP binding loop fluctuations with (C80-SNO) or without (C80-SH) *S*-nitrosylation modification at Cys^{80} at start and end of the simulations. (**C**) Root means square deviation (RMSD) plot of BIK1 throughout the 80 ns MD simulation. (**D**) Root mean squared fluctuation (RMSF) plot per residue of the BIK1 during the MD simulation. (E) Rosette morphology of independent *BIK1^{C80A}/bik1* lines at 4 weeks after germination grown in short-day conditions. (F) BIK1 kinase activity was determined *in vitro* by examining its autophosphorylation. Recombinant MBP-BIK1 or MBP-BIK1^{C80A} was subjected to a kinase activity assay. CBB staining was employed as a loading control.

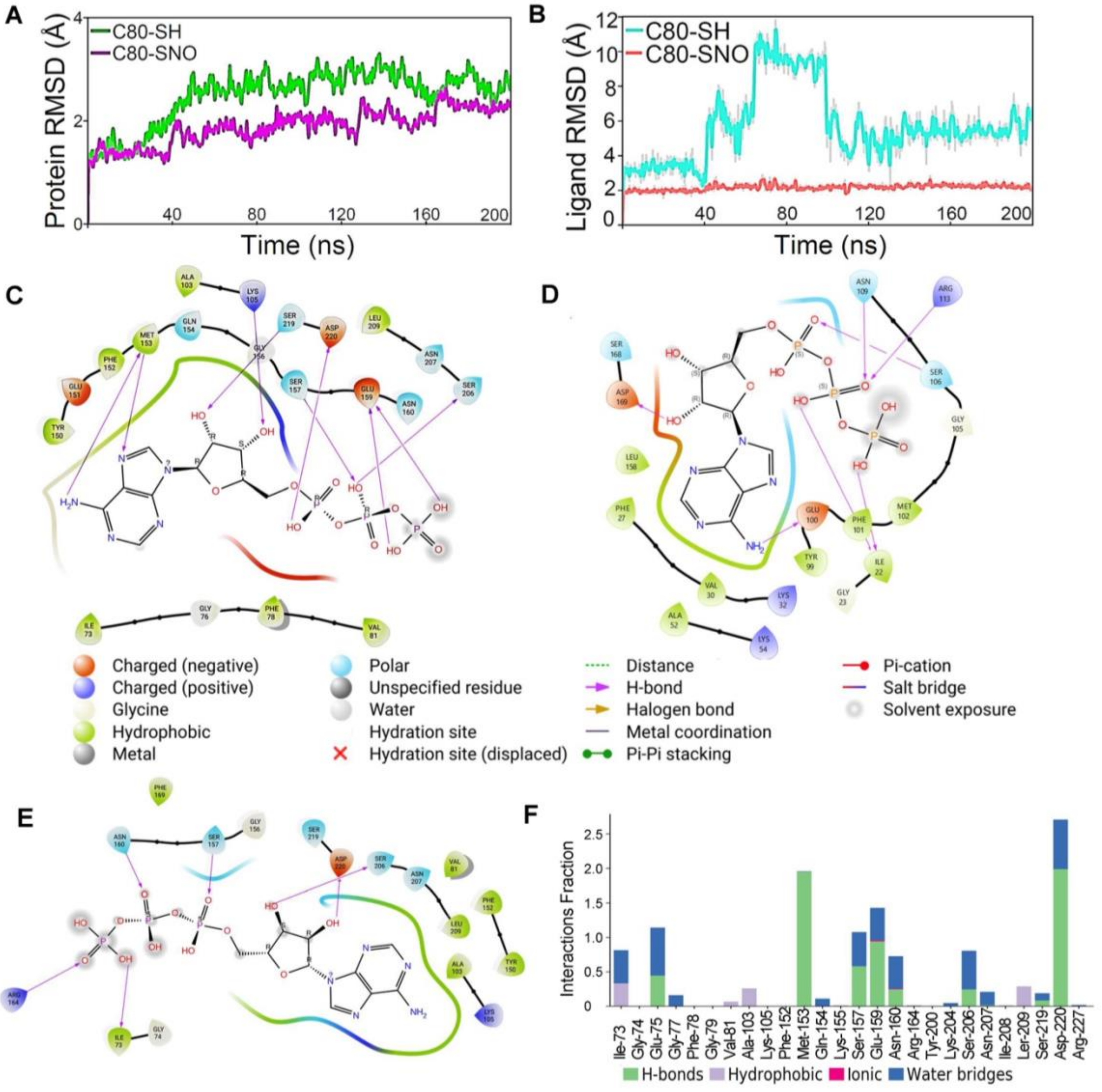

Fig. S13 BIK1 Cys^{80}-SNO formation increases BIK1 ATP affinity.

(**A-B**) *S*-nitrosylation of BIK1 at Cys^{80} increases the stability of both BIK1 (A) and its ATP ligand (B). (**C-E**) Comparison of residue interaction networks between C80-SNO (C) or C80A (D), C80-SH (E) with ATP. *S*-nitrosylation of residue Cys^{80} of BIK1 enhanced interaction with ATP which is otherwise present in the NO modified BIK1 in reduced association with the ligand. Docking score: C80-SNO, -9.434 kcal/mol; C80A, -7.024 kcal/mol; C80-SH, -7.000 kcal/mol. (**F**) The residues that interact with ATP in BIK1-C80-SNO.

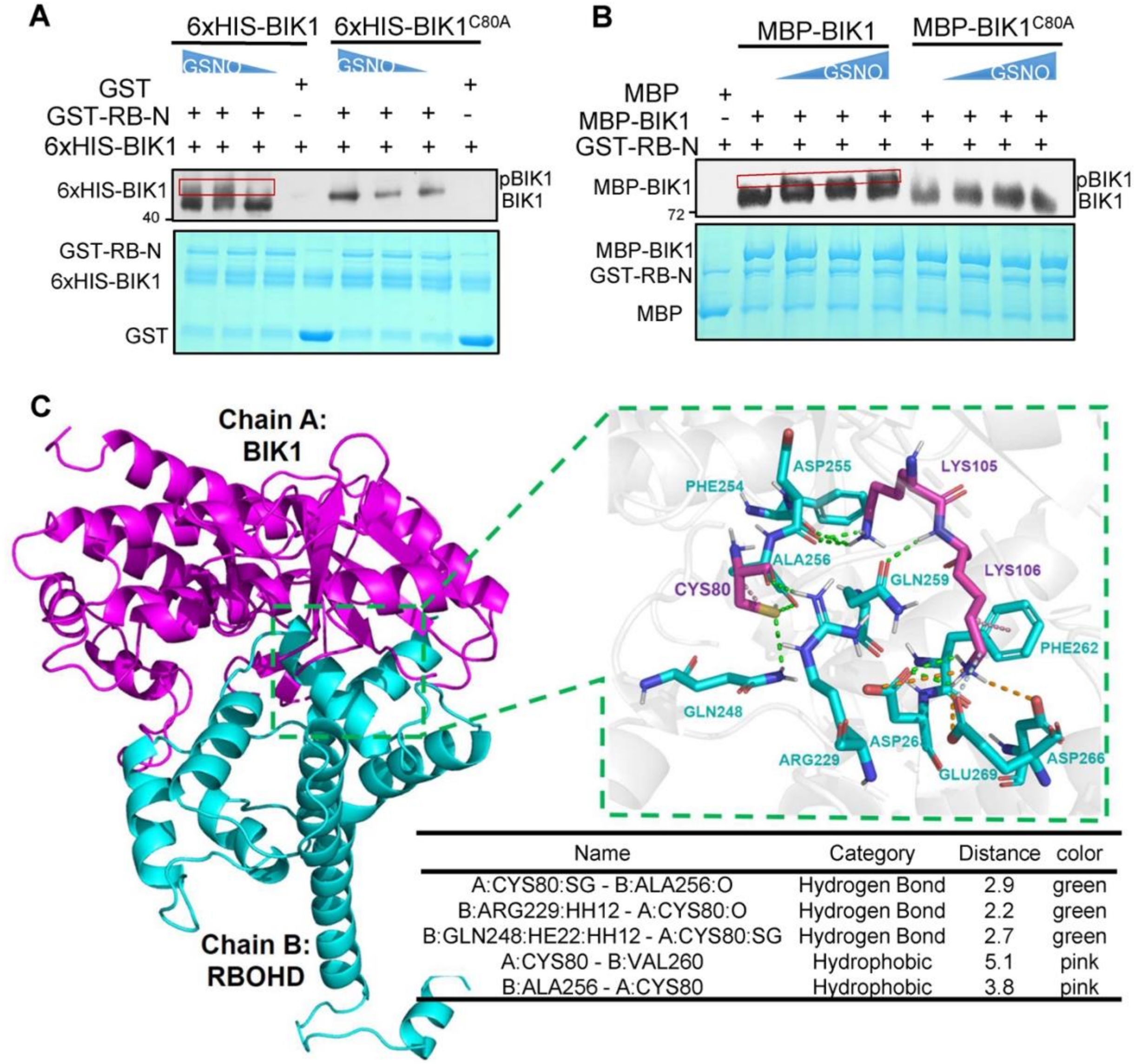

Name	Category	Distance	color
A:CYS80:SG - B:ALA256:O	Hydrogen Bond	2.9	green
B:ARG229:HH12 - A:CYS80:O	Hydrogen Bond	2.2	green
B:GLN248:HE22:HH12 - A:CYS80:SG	Hydrogen Bond	2.7	green
A:CYS80 - B:VAL260	Hydrophobic	5.1	pink
B:ALA256 - A:CYS80	Hydrophobic	3.8	pink

Fig. S14 *S*-nitrosylation of BIK1 at Cys^{80} promotes interaction with RBOHD.

(**A-B**) Glutathione S-transferase (GST) or GST-RBOHD N-terminus (GST-RB-N) immobilized on glutathione Sepharose beads were incubated with either HIS-BIK1, MBP, MBP-BIK1 or associated variant proteins and subsequently subjected to GST pull-down assays. Phosphorylated BIK1 (pBIK1) levels were detected by mobility-shift assays and are indicated within the red pane. Total protein loading is shown by coomassie brilliant blue (CBB) staining. (**C**) Computer modeling to show the interacting surface between BIK1 and RBOHD. Chain A, BIK1, purple. Chain B, RBOHD N terminus, cyan. Chemical bonds between BIK1-CYS80 and RBOHD amino acid residues are listed.

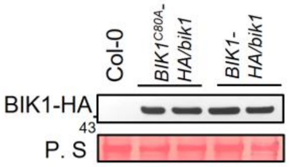

Fig. S15 Detection of BIK1-HA levels in transgenic lines utilized in fig. 4 E-F.

Total protein extracted from representative lines or wild-type Col-0 were subjected to a western blotting assay. Ponceau S. (P. S) staining served as a loading control.

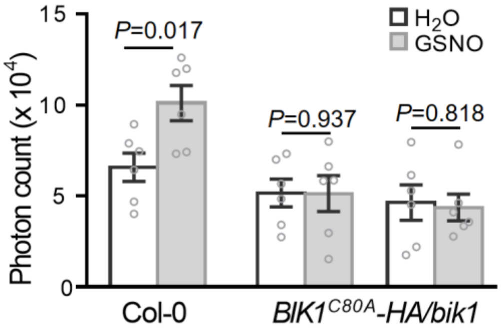

Fig. S16 Precluding *S*-nitrosylation of BIK1 at Cys[80] inhibits both elf18 and GSNO induced ROS production.

Leaf discs from indicated plants were treated with 200 nM elf18, along with either H_2O or 10 μM GSNO. Subsequently, the total ROS burst was calculated over 60 mins. Values are mean ± SE, n=6. one-way ANOVA.

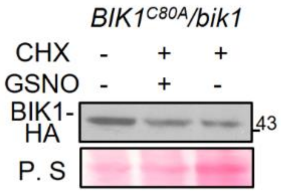

Fig. S17 Precluding *S*-nitrosylation of BIK1 at Cys[80] decreases BIK1 stability.

BIK1^{C80A}-HA expressing plants were treated with 100 μM cycloheximide (CHX) and 10 μM GSNO for 8 hours before total protein was examined with immunoblot. Ponceau S. (P. S) indicates equal loading.

Table S1 Identification of the *S*-nitrosylation site of BIK1 by mass spectrometry.

Peptide	Cysteine position	Treatment	Peptide coverage	Total PSMs	C(Carbamidomethyl)	C(Nethyl maleimide	Labling efficiency
NFRPDSVIGEGGFG**C**VFK	C80	GSH	76.96%	2	2	0	0.00%
		GSNO	84.81%	2	2	0	0.00%
MASVAVQ**C**LSFEPK	**C331**	GSH	76.96%	60	48	12	20.00%
		GSNO	84.81%	84	69	15	17.85%
LIGY**C**LEDEHR	**C140**	GSH	76.96%	28	14	14	50.00%
		GSNO	84.81%	32	23	9	28.12%
MGS**C**FSSR	C4	GSH	75.19%	8	6	2	25.00%
		GSNO	59.75%	35	35	0	0.00%

Table S2 Complex binding free energy (kcal/mol) calculated by the MM/GBSA (molecular mechanics energies combined with the Poisson–Boltzmann or generalized Born and surface area continuum solvation) algorithm. Bind**,** Binding energy; Coulomb, Covalent binding energy; Hbond, hydrogen-bonding correction; Lipo, lipophilic energy; vdW, van der Waals energy.

Complex	G_Bind	ΔG_Coulomb	Δ_Hbond	ΔG_Lipo	ΔG_vdW
C80-SH	-45.11	-65.97	-2.98	-6.51	-41.6
C80-SNO	-68.56	-48.11	-5.61	-10.34	-46.55

Table S3 List of primers used in this study.

Gene name	Sense primer (5'-3')	Antisense primer (5'-3')
**Quantitative RT-PCR**		
BIK1	ACTTATGGGTACGCCGCGCCTGAGT	GGCACGGACCACTTGGTCCA
BAK1	ACCGCCTCCTATCTCTCCTACACC	CTGGGTCCTCTTCAGCTGGTACA
FRK1	CGGTCAGATTTCAACAGTTGTC	AATAGCAGGTTGGCCTGTAATC
FLS2	AGGGTTTGCGTGGGAAAG	GACATGGTTTTCTATCAGTCTA
UBQ10	AGATCCAGGACAAGGAAGGTATTC	CGCAGGACCAAGTGAAGAGTAG
Actin	AATCCACGAGACAACCTA	AGCGATACCTGAGAACATA
**Cloning**		
BIK1	GGGGACAAGTTTGTACAAAAAAGCAGGCTTC ATGGGTTCTTGCTTCAGTTCTCG	GGGGACCACTTTGTACAAGAAAGCTGGGTCT CTTGGACCCGAGGGGTATTC
RBOHD-N	GGGGACAAGTTTGTACAAAAAAGCAGGCTTC ATGAAAATGAGACGAGGCAATTC	GGGGACCACTTTGTACAAGAAAGCTGGGTCT CTCTGCCAATTGTCAAGTATGA
BIK1 promoter	GGGGACAAGTTTGTACAAAAAAGCAGGCTTC GTGGGCATAAACTATAACACCT	CAAAGCTAAGAACAGATTCGTT
**Point mutation**		
BIK1-C80A	CGGAGAAGGTGGCTTTGGTGCTGTCTTTAAA GGCTGG	CCAGCCTTTAAAGACAGCACCAAAGCCACCT TCTCCG
BIK1-C140A	AATCTAGTTAAACTGATCGGTTATGCCTTAGA AGATGAACACCGTCTTC	GAAGACGGTGTTCATCTTCTAAGGCATAACC GATCAGTTTAACTAGATT
BIK1-C331A	AAGCGTGGCGGTGCAGGCTCTCTCATTTGAA CCC	GGGTTCAAATGAGAGAGCCTGCACCGCCAC GCTT
BIK1-C4A	AGCAGGCTTAATGGGTTCTGCCTTCAGTTCTC GAGTCAAA	TTTGACTCGAGAACTGAAGGCAGAACCCATT AAGCCTGCT
**Genotyping**		
bik1	TACTTGGGGCAACTGAGTCAC	TTTGTGTAAACCGTTTTTCGG
fls2	GGAGACAGAACACCTTCAAGT	TGACCAGATTCCTCAATAGTC
LBb1.3	ATTTTGCCGATTTCGGAAC	
